# Recurrence-associated pathways in hepatitis B virus-positive hepatocellular carcinoma

**DOI:** 10.1186/s12864-015-1472-x

**Published:** 2015-04-10

**Authors:** Bu-Yeo Kim, Dong Wook Choi, Seon Rang Woo, Eun-Ran Park, Je-Geun Lee, Su-Hyeon Kim, Imhoi Koo, Sun-Hoo Park, Chul Ju Han, Sang Bum Kim, Young Il Yeom, Suk-Jin Yang, Ami Yu, Jae Won Lee, Ja June Jang, Myung-Haing Cho, Won Kyung Jeon, Young Nyun Park, Kyung-Suk Suh, Kee-Ho Lee

**Affiliations:** Division of Radiation Cancer Research, Korea Institute of Radiological and Medical Sciences, 215-4 Gongneung-dong, Nowon-ku, Seoul, 139-706 Korea; Herbal Medicine Research Division, Korea Institute of Oriental Medicine, Daejeon, Korea; Department of Pathology, Korea Institute of Radiological and Medical Sciences, Seoul, Korea; Department of Internal Medicine, Korea Institute of Radiological and Medical Sciences, Seoul, Korea; Department of Surgery, Korea Institute of Radiological and Medical Sciences, Seoul, Korea; Department of Bioinformatics and Biostatistics, University of Louisville, Louisville, USA; Medical Genomics Research Center, Korea Research Institute of Bioscience and Biotechnology, Daejeon, Korea; Department of Statistics, Korea University, Seoul, Korea; Department of Pathology, Seoul National University College of Medicine, Seoul, Korea; Department of Surgery, Seoul National University College of Medicine, Seoul, Korea; Laboratory of Toxicology and Research Institute for Veterinary Science and College of Veterinary Medicine, Seoul National University, Seoul, Korea; Department of Surgery, Samsung Medical Center, Sungkyunkwan University School of Medicine, Seoul, Korea; Department of Pathology and BK 21 PLUS Project for Medical Science, Yonsei University College of Medicine, Seoul, Korea; Korean Medicine Clinical Trial Center, Kyung Hee University Oriental Medicine Hospital, Seoul, Korea

**Keywords:** Recurrence-associated pathway, Hepatocellular carcinoma, Principal component analysis, Prognosis, Risk, Small tumor

## Abstract

**Background:**

Despite the recent identification of several prognostic gene signatures, the lack of common genes among experimental cohorts has posed a considerable challenge in uncovering the molecular basis underlying hepatocellular carcinoma (HCC) recurrence for application in clinical purposes. To overcome the limitations of individual gene-based analysis, we applied a pathway-based approach for analysis of HCC recurrence.

**Results:**

By implementing a permutation-based semi-supervised principal component analysis algorithm using the optimal principal component, we selected sixty-four pathways associated with hepatitis B virus (HBV)-positive HCC recurrence (*p* < 0.01), from our microarray dataset composed of 142 HBV-positive HCCs. In relation to the public HBV- and public hepatitis C virus (HCV)-positive HCC datasets, we detected 46 (71.9%) and 18 (28.1%) common recurrence-associated pathways, respectively. However, overlap of recurrence-associated genes between datasets was rare, further supporting the utility of the pathway-based approach for recurrence analysis between different HCC datasets. Non-supervised clustering of the 64 recurrence-associated pathways facilitated the classification of HCC patients into high- and low-risk subgroups, based on risk of recurrence (*p* < 0.0001). The pathways identified were additionally successfully applied to discriminate subgroups depending on recurrence risk within the public HCC datasets. Through multivariate analysis, these recurrence-associated pathways were identified as an independent prognostic factor (*p* < 0.0001) along with tumor number, tumor size and Edmondson’s grade. Moreover, the pathway-based approach had a clinical advantage in terms of discriminating the high-risk subgroup (N = 12) among patients (N = 26) with small HCC (<3 cm).

**Conclusions:**

Using pathway-based analysis, we successfully identified the pathways involved in recurrence of HBV-positive HCC that may be effectively used as prognostic markers.

**Electronic supplementary material:**

The online version of this article (doi:10.1186/s12864-015-1472-x) contains supplementary material, which is available to authorized users.

## Background

Various gene signatures related to the survival and recurrence of hepatocellular carcinoma (HCC) have been identified using microarray analysis, and proposed to supplement clinicopathological prognostic factors for prediction of patient outcomes [1-4]. In fact, prognostic genes are beneficial for the development of risk scores based on gene expression, that can overcome the limitations of clinical prognostic factors [5,6]. Interestingly, prognostic genes can be extracted from not only HCC, but also adjacent non-tumor liver tissues [7,8]. However, the heterogeneous clinicopathological and biological nature of HCCs makes it difficult to identify significant common genes that fit to different datasets [7,9]. Therefore, elucidation of the common biological functions related to prognosis that may be applicable to different datasets is also difficult.

One way to overcome the limitations of the gene-based approach is the use of functionally related predefined gene sets, such as pathways, instead of individual genes [10,11]. Pathway analysis provides biological information that facilitates characterization of the functional network and the relationships between selected significant genes [12]. A number of methods have been proposed to identify the pathways associated with prognosis [10,13]. Recent reports also indicate that rather than whole genes, a subset of genes correlated with sample phenotype is more accurate in predicting clinical outcomes of patients [14].

Among the diverse factors implicated in HCC development, hepatitis B (HBV) and hepatitis C viruses (HCV) are the major etiological risk factors. Although HCCs infected chronically with HBV and HCV are not distinguishable from histological and clinical evaluations [15], studies to date consistently imply that different molecular mechanisms underlie the development of HBV- and HCV-positive HCCs [16,17]. We additionally observed functional differences and similarities between the development of HCV- and/or HBV-positive HCC at the pathway level [12]. These results strongly suggest differences in biological behavior and consequent responses to treatment between HBV and HCV-positive HCCs.

In the present study, we implemented the pathway-based supervised principal component analysis (PCA) with random permutations to identify recurrence-associated pathways of HBV-positive HCC. Comparison of our HBV-positive recurrence-associated pathways with those from two public datasets of HBV and HCV positive-HCCs revealed significant overlap of pathways, despite limited common genes between the datasets. The set of recurrence-associated pathways identified in this study effectively facilitated the stratification of HBV-positive HCC patients according to risk of recurrence. Our pathway-based approach should therefore provide clinically useful molecular insights into the mechanisms underlying HCC recurrence.

## Results

**Figure 1:**
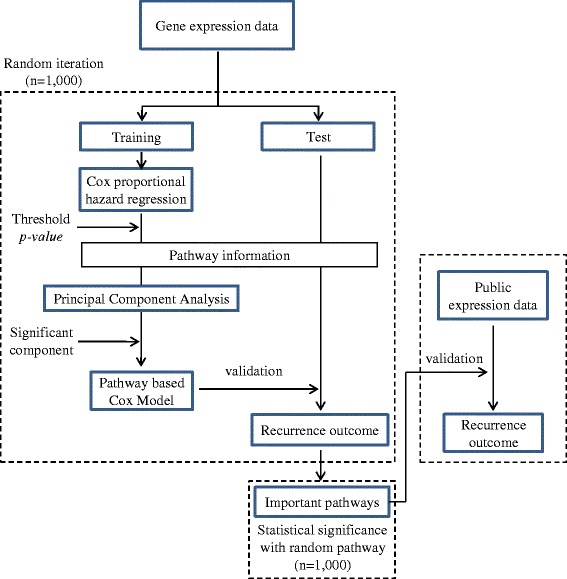
Schematic illustration of the analysis strategy. The initial dataset was randomly divided into training and test sets. In a training set, genes below the threshold *p-*value in a pathway were subjected to PCA. Two models were constructed, specifically, one principal component model using the most significantly associated principal component and weighted model using multiple principal components, and evaluated in the test set. This procedure was repeated 1,000 times with random training and test sets. Finally, median values of statistics from test sets were measured to select significant pathways.

To determine the biological pathways associated with HCC prognosis, we established a genome-wide gene expression dataset via cDNA microarray of 142 HCC cases positive for HBV. A schematic diagram of the overall procedure for selection of pathways associated with recurrence is presented in Figure 1.

### Identification of genes related to HCC recurrence

**Figure 2:**
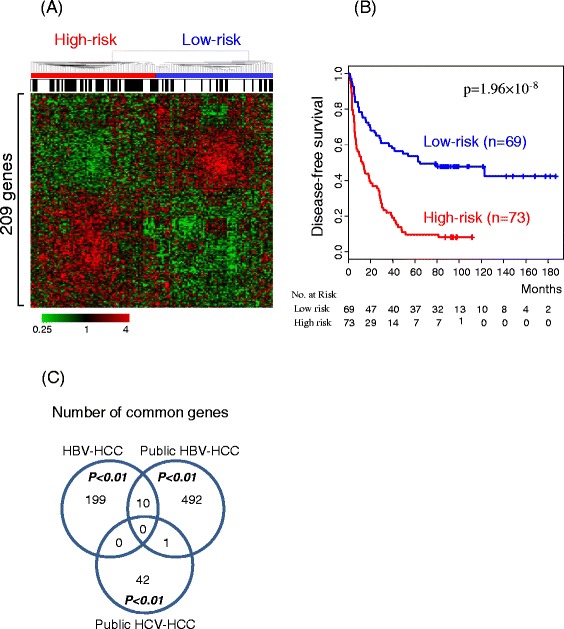
Cluster analysis of recurrence-related genes. **(A)** Dendrogram of clustering pattern measured from the matrix of 209 recurrence-associated genes (Cox regression *p-*value < 0.01) from our HBV-HCC. Samples were classified into two subgroups: low-risk and high-risk based on recurrence outcome. The black bar indicates patients with early recurrence within 2 years after surgery. Columns represent individual samples, and rows genes. Red and green colors reflect high and low expression levels, respectively, as indicated by scale bars. **(B)** Kaplan-Meier plots for recurrence rates of the low- and high-risk subgroups. *P*-values were obtained using the log-rank test. **(C)** Overlap of recurrence-associated genes (Cox regression *p-*value < 0.01) among datasets.

Initially, using the microarray dataset and long-term follow-up clinical data, we assessed the implications of gene expression on HCC recurrence. In our cohort, median overall and recurrence free-survival times were 73.0 and 26.5 months, respectively. As an initial attempt, we extracted a subset of 209 genes correlated with HCC recurrence (log rank *p-*value < 0.01) (Additional file 1: Table S1). When these recurrence-associated genes were subjected to the unsupervised clustering method [7,14], their hierarchical clustering led to the discrimination of HCC patients into low-risk and high-risk subclasses (Figure 2A). Kaplan-Meier plot of recurrence-free survival based on expression of the 209 recurrence-associated genes (Figure 2B) showed significant discrimination of HCC patients according to risk, in which median recurrence-free survival rates of low (N = 69) and high-risk (N = 73) subgroups were 64.6 and 13.0 months (log rank *p-value* <0.0001), respectively. Upon further differentiation of these patients based on 2 years after surgery, defined as a cut-off for early recurrence [18,19], those showing recurrence within the 2-year period were classified into the high-risk subgroup.

To further evaluate the expression profiles for recurrence-free survival, we compared recurrence-associated genes of our current dataset with those from two public HCC datasets with available recurrence information, specifically, a HBV-positive HCC dataset (GSE14520, termed ‘public HBV-HCC’) [3] and HCV-positive HCC dataset (GSE17856) (‘public HCV-HCC’) [4]. In total, 503 and 43 genes from these two public datasets were selected, respectively (log rank *p-*value < 0.01). Comparison of the recurrence-related genes between datasets (209, 503 and 43 genes from our HBV-HCC, public HBV-HCC and HCV-HCC datasets, respectively) disclosed a limited number of common genes (Figure 2C). Only ten genes overlapped between our HBV-HCC and public HBV-HCC despite common viral etiology. Moreover, no common genes were identified among the three datasets. Indeed, the lack of common genes among different microarray datasets is the most significant problem in analysis of prognosis of tumors, including HCC [9,20].

### Comparison of clinical distribution in HCC datasets

**Figure 3:**
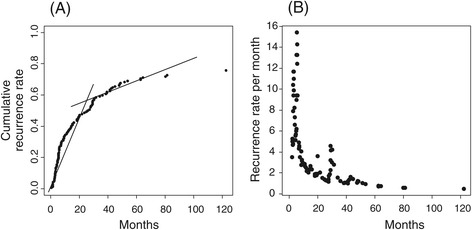
Recurrence rates in the HBV-HCC dataset. **(A)** Cumulative recurrence rate of HCC over time. **(B)** Recurrence rate of HCC per month over time.

The distributions of clinical variables in the three datasets, including ours, are presented in Additional file 2: Table S2. Owing to the lack of specific variables, such as tumor differentiation and fibrosis, in the public HBV-HCC dataset, comparison of the datasets was difficult. In addition, tumor stage (available from all three datasets) was different among datasets. Specifically, the proportion of advanced-stage tumors III and IV in our HBV-HCC dataset was 34.5%, compared to those of public HBV-HCC (22.8%) and HCV-HCC datasets (25.6%) (*p-*value < 0.0001). However, the incidence of recurrence in our HBV-HCC dataset showed a biphasic pattern (Figure 3A), similar to the public HBV-HCC dataset [3]. Specifically, the cumulative recurrence rate was 33.80% per year during the first two years after surgical resection of tumor in our HBC-HCC, whereas the rate decreased to 4.01% per year beyond two years after surgery (Figure 3A). In agreement with this result, the recurrence rate per month peaked during the first two years and persisted through the following years (Figure 3B). In addition, tumor differentiation and fibrosis stages were similar between our dataset and public HCV-HCC, indicating clear advantages in comparing expression patterns and pathways associated with recurrence.

### Identification of recurrence-associated pathways between different HCC datasets

**Figure 4:**
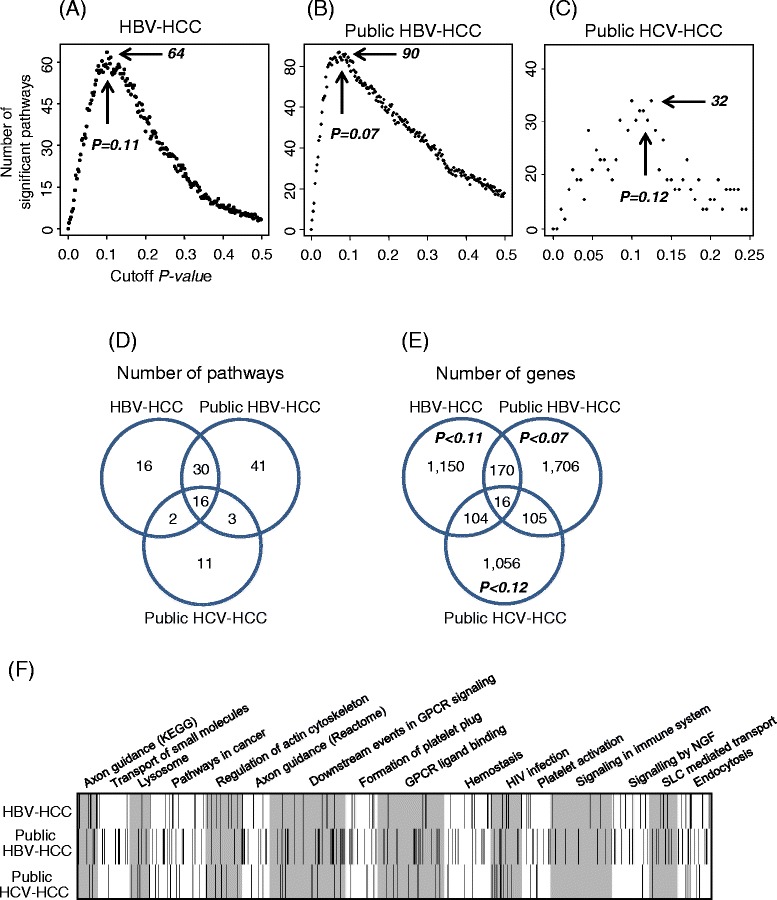
Determination of threshold *p-*value and comparison of recurrence-related features among the three HCC datasets. Starting with gene of the lowest univariate Cox *p-*value, permutation-based pathway analysis was applied by gradually increasing the *p-*value. The maximum average number of pathways below *p-*value of 0.01 in the test set was measured at threshold *p-*values of 0.11, 0.07 and 0.12 from **(A)** HBV-HCC, **(B)** public HBV-HCC and **(C)** public HCV-HCC, respectively, as indicated with arrows. **(D)** Overlap of recurrence-associated pathways obtained at threshold *p-*values among datasets. **(E)** Overlap of recurrence-associated genes at threshold *p-*values among datasets. **(F)** Distribution of genes below the threshold *p-*value from each dataset in 16 common significant pathways.

To overcome the limitations of the gene-based approach, we applied a pathway-based semi-supervised PCA approach. Following the overall analytical procedure depicted in Figure 1, we initially determined a threshold *p*-value that maximally generated significant pathways in each dataset. Based on plots showing changes in the number of significant pathways when the *p*-value is gradually increased, threshold *p*-value were determined as 0.11, 0.07 and 0.12, and among a total of 882 pathways, we subsequently obtained 64, 90 and 32 pathways of maximum significance (log-rank *p*-value < 0.01 in the test set) for our HBV-HCC, public HBV-HCC and public HCV-HCC datasets, respectively (Figure 4A-C). Among the 64 significant pathways identified within our HBV-HCC dataset, 46 (71.9%) and 18 (28.1%) were in common with 90 and 32 significant pathways of public HBV-HCC and HCV-HCC datasets, respectively (Figure 4D). In contrast to pathway-based analysis showing significant overlap between the datasets, the gene-based approach revealed a low rate of overlap of significant genes (12.9% and 8.3%, respectively) under conditions of the same threshold *p-*values (Figure 4E). Moreover, the majority of significant genes from each dataset were not commonly distributed on the 16 overlapping pathways in all three datasets (Figure 4F; also refer to the significant pathways from each dataset in Additional file 3: Table S3).

Comparison of the significant pathways extracted from one optimal PCA method with those from weighted PCA (see Methods) disclosed similar results (Additional file 4: Figure S1). Interestingly, signaling pathways related to cell proliferation and invasiveness, such as mitogen-activated protein kinase (MAPK), ErbB, G protein-coupled receptor (GPCR), and membrane integrity-related pathways, such as those involving actin cytoskeleton, axon guidance and adhesion, were significantly associated with recurrence in our HBV-HCC dataset (Figure 4F and Additional file 3: Table S3). Moreover, all significant pathways obtained from each dataset (log-rank *p*-value < 0.01 in the test set) were associated with early recurrence within two years, with area under curve (AUC) > 0.6. For example, in Kaplan-Meier plots and receiver operating characteristic (ROC) curves of the axon guidance pathway, one significant pathway common to all datasets clearly distinguished patients according to recurrence risk, with AUC > 0.65 (Additional file 5: Figure S2).

### Subgroup classification via semi-supervised clustering of pathways

**Figure 5:**
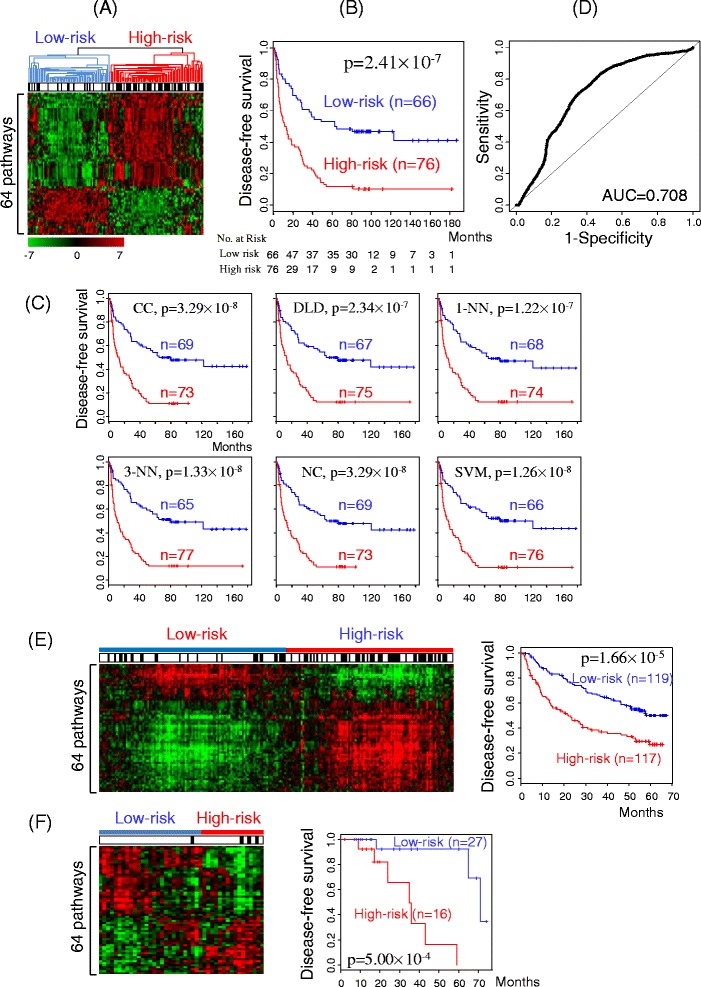
Cluster analysis of recurrence-related pathways. **(A)** Dendrogram of the clustering pattern measured from the matrix of principal components of 64 recurrence-associated pathways (*p-*value < 0.01) from HBV-HCC. Samples were classified into two subgroups: low-risk and high-risk. The black bar indicates patients with early recurrence within 2 years after surgery. Columns represent individual samples, and rows pathways. Red and green colors reflect high and low levels of optimal principal component scores, respectively, as indicated by scale bars. **(B)** Kaplan-Meier plots for recurrence rates of low- and high-risk subgroups. *P*-values were obtained using the log-rank test. **(C)** Cross-validation of the low- and high-risk subgroups using six different algorithms: compound covariate (CC), diagonal linear discriminant (DLD), 1-nearest neighbor (1-NN), 3-nearest neighbor (3-NN), nearest centroid (NC) and support vector machine (SVM), implemented in BRB ArrayTools. **(D)** ROC curve computed with the compound covariate validation algorithm for subgroup classification. **(E and F)** Dendrogram of the clustering pattern measured from the matrix of principal components of public HBV-HCC **(E)** or public HCV-HCC **(F)** on the 64 pathways associated with recurrence in our HBV-HCC dataset.

To utilize individual pathway information, sample scores obtained from optimal principal components of the 64 recurrence-associated pathways were hierarchically clustered using unsupervised clustering analysis of gene expression [7,14], as depicted in Figure 2. Figure 5A shows the resulting two subgroups of HCC patients with different outputs of recurrence (log rank *p-*value < 0.0001 in Figure 5B). Similar to the gene expression-based approach, the pathway-based assay classified patients with early recurrence within 2 years after surgery predominantly in the high-risk, relative to the low-risk subgroup. The robustness of the two subgroups was validated using leave-one-out cross-validation with six algorithms, including compound covariate, diagonal linear discriminant analysis, 1-nearest neighbor, 3-nearest neighbor, nearest centroid, and support vector machine. The algorithms clearly divided the high- and low-risk subgroups with cross-validated misclassification error rate below 0.1 and corresponding *p-*values < 0.0001, based on 1,000 random permutations (Figure 5C). ROC curve computed with compound covariate algorithm values from subgroup classification using the principal components matrix showed an AUC value of 0.708 for early recurrence with sensitivity and specificity of 0.72 and 0.64, respectively (Figure 5D). Similarly, 90 and 32 recurrence-associated pathways obtained from the public HBV-HCC and HCV-HCC datasets, respectively, clearly discriminated high-risk and low-risk recurrence subgroups (*p-*value < 0.0001) (Additional file 6: Figure S3A ~ C and Additional file 7: Figure S4A ~ C). ROC curves additionally showed that these pathways are significantly associated with early recurrence in both datasets (Additional file 6: Figure S3D and Additional file 7: Figure S4D). Our findings indicate that combining the recurrence-associated pathways increases the statistical significance of patient classification according to recurrence risk.

Since our approach using optimal principal components was dependent on data structure, such as microarray platform and experimental conditions, we could not directly apply coefficients obtained from our microarray dataset to the other independent microarray datasets. Therefore, only pathway information from our HBV-HCC dataset was used to validate the effectiveness of pathway analysis in the public microarray datasets. Using our 64 recurrence-associated pathways, sample scores from the first, not optimal, principal component were measured and clustered in public HBV (Figure 5E) and public HCV datasets (Figure 5F). As a result, two subgroups (low- and high-risk) were assigned, depending on the recurrence outcome. As expected from the significant number of common pathways among the three datasets (Figure 4D), these 64 pathways from our HBV-HCC dataset were also effective in discriminating patients of public microarray datasets according to risk (log rank *p-*value < 0.001).

### Clinical association of recurrence-related pathways

**Figure 6:**
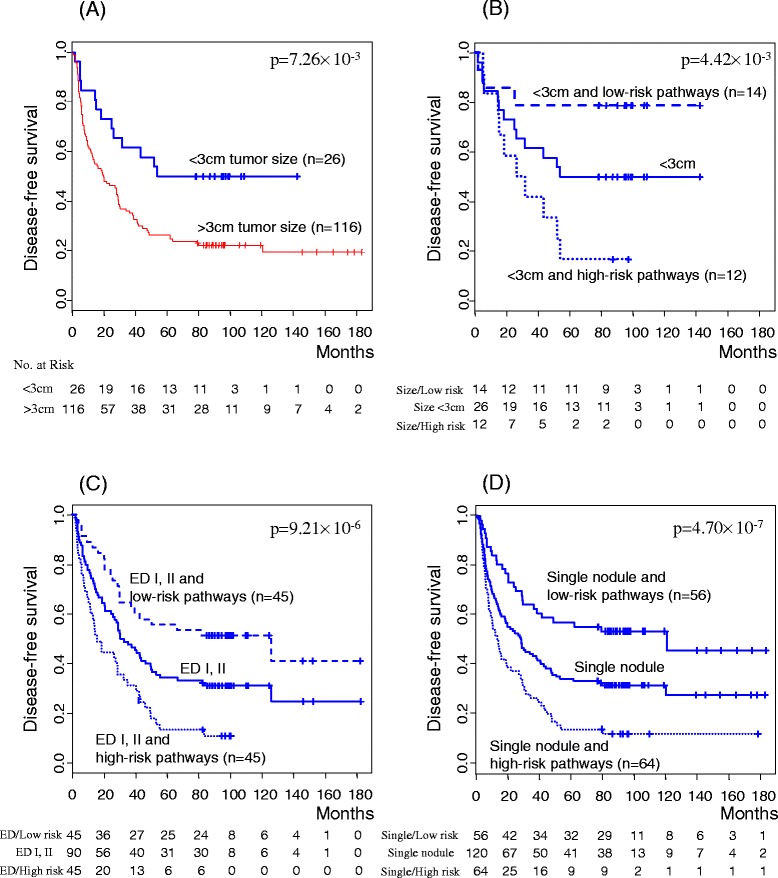
**Stratification of small tumor size patients by recurrence-associated pathways. (A)** Kaplan-Meier plot for recurrence rates in two subgroups of patients based on tumor size (< and >3 cm) in our HBV-HCC dataset. *P*-values were obtained using the log-rank test. **(B-D)** Application of recurrence-associated pathways to patients with small tumors (<3 cm) **(B)**, Edmonson Steiner’s grade (I and II) **(C)** or single nodules **(D)** led to further stratification into two subgroups with different recurrence rates.

Next, we examined the clinical implications of the recurrence-associated 64 pathways in our HBV-HCC dataset (Table 1). Clinicopathological variations (*p-*value < 0.05) associated with the high-risk subgroup classified according to pathway analysis included high levels of aspartate aminotransferase (≥40 IU/L) and alanine aminotransferase (≥40 IU/L), and presence of vascular invasion and lobular activity. Absence of capsule and narrow resection margin (<2 cm) were also marginally associated with the high-risk subgroup (*p-*value < 0.1). Univariate analysis showed that the 64 pathways (*p-*value < 0.0001), together with clinicopathological parameters, including tumor stage (*p-*value = 0.0404), tumor number (*p-*value = 0.0125), tumor size (*p-*value = 0.0072), vascular invasion (*p-*value = 0.0059), lobular activity (*p-*value = 0.0065), Child-Pugh score (*p-*value = 0.0596), Edmonson Steiner’s grade (*p-*value = 0.0764) and capsule formation (*p-*value = 0.0560), are significantly or marginally associated with HCC recurrence in our present cohort. Multivariate analysis further led to the definition of these pathways (*p-*value < 0.0001) as an independent prognostic factor for recurrence together with clinical variables, including tumor number (*p-*value = 0.0212), tumor size (*p-*value = 0.0066) and Edmondson Steiner’s grade (*p-*value = 0.0082), in our HBV-positive HCC dataset. In particular, focusing on tumor size among the variables (Figure 6A), the recurrence-associated 64 pathways led to the extraction of a subgroup showing poor prognosis from patients with small tumors (<3 cm) in our HBV-HCC dataset (Figure 6B). Similarly, recurrence-associated pathways in the public HBV-HCC dataset facilitated the identification of a subgroup of early recurrence in patients with small tumors (<5 cm) (Additional file 8: Figure S5). In addition, Edmondson Steiner’s grade (Figure 6C) and tumor number (Figure 6D), significant parameters in multivariate analysis (Table 1), were effective in the extraction of a subgroup with early recurrence from patients with Edmondson Steiner’s grade I and II or single nodular status. These findings further validate the clinical utility of our pathway-based approach when combined with other clinical variables.

**Table 1:** **Relationships between recurrence-associated pathways and clinicopathological variables in HBV-HCC**

	**Pathways-based subgroups**			**Recurrence analysis**	
				**Univariate analysis**	
**Variables**	**Low risk**	**High risk**	**P-value** ^**1**^	**Hazard ratio (95% CI)** ^**2**^	**Log rank**
					**p-value** ^**3**^
Gender (Male/Female)	52/14	65/11	0.406	0.563 (0.320 0.991)	**0.0439**
Age (<50 y/≥50 y)	26/40	27/49	0.763	1.056 (0.706 1.577)	0.790
Platelet (<170 × 10^9^/L/≥170 × 10^9^/L)	33/25	28/36	0.204	1.019 (0.675 1.538)	0.928
^4^AST (<40 IU/L/≥40 IU/L)	40/26	31/45	**0.0287**	1.096 (0.745 1.613)	0.639
^4^ALT (<40 IU/L/≥40 IU/L)	41/25	33/43	**0.0397**	1.003 (0.682 1.477)	0.984
Bilirubin (<1 mg/dL/≥1 mg/dL)	44/22	42/34	0.224	1.050 (0.708 1.556)	0.806
^4^AFP (<300 ng/mL /≥300 ng/mL)	41/25	52/23	0.469	0.976 (0.644 1.480)	0.912
Child-Pugh (A/B,C)	63/3	65/10	0.131	1.813 (0.967 3.401)	0.0596
UICC (I,II/III,IV)	39/25	50/22	0.389	1.519 (1.015 2.273)	**0.0404**
Size (<3 cm/≥3 cm)	14/52	12/64	0.538	2.178 (1.216 3.901)	**0.00726**
Number (Single/Multiple)	56/10	64/10	0.972	1.874 (1.135 3.094)	**0.0125**
Necrosis (No/Yes)	33/33	25/50	0.0664	1.565 (1.048 2.339)	**0.0273**
Edmondson’s grade (I,II/III,IV)	45/19	45/27	0.435	1.273 (0.974 1.665)	0.0764
Vein invasion (Absent/Present)	49/12	39/31	**0.00501**	1.787 (1.175 2.718)	**0.00594**
Lobular activity (No/Yes)	39/25	18/56	**2.879 × 10** ^**−5**^	1.770 (1.166 2.685)	**0.00652**
Capsule (Absent/Present)	47/19	64/11	0.0660	0.617 (0.374 1.017)	0.05603
Margin (<2 cm/≥2 cm)	35/31	51/23	0.0794	0.836 (0.557 1.256)	0.389
Cirrhosis (Absent/Present)	37/29	41/34	0.997	1.257 (0.853 1.852)	0.245
Recurrence-associated pathways	-	-	-	2.843 (1.881 4.295)	**2.416 × 10** ^**−7**^
				**Multivariate analysis**	
Recurrence-associated pathways				3.220 (2.076 4.994)	**1.760 × 10** ^**−7**^
Number (Single/Multiple)				1.839 (1.095 3.089)	**0.0212**
Size (<3 cm/≥3 cm)				2.489 (1.288 4.808)	**0.00666**
Edmondson’s grade (I,II/III,IV)				1.769 (1.159 2.701)	**0.00821**

^1^ *p-*values were calculated using χ^2^ test.

^2^CI represents confidence interval.

^3^ *p-*values were calculated using the Cox Proportional Hazards Model.

^4^AST, aspartate aminotransferase; ALT, alanine aminotransferase; AFP, α-fetoprotein.

Bold data indicate statistically significant values (*p*<0.05).

## Discussion

The identification of common biological features associated with HCC prognosis based on gene expression patterns that are interchangeable among different patient groups is a challenge, since shared significant prognostic genes among patient cohorts are extremely limited. As expected, common significant recurrence-associated genes were rare among the HCC datasets examined in the present study, with only 4.8% overlapping genes identified between the two HBV-positive HCC groups. Several factors may contribute to the observed rarity of common genes. Heterogeneity of patients in terms of clinicopathological characteristics as well as differences in microarray platforms and experimental conditions are among the main causes of inconsistent microarray results among datasets. A gene set-based approach (pathway analysis) was recently shown to partly overcome the heterogeneity of microarray analysis [10,13,14]. The use of biologically related gene sets may be a more robust approach to suppress clinicopathological heterogeneity [12].

Data from the present study confirmed that the pathway-based approach is more beneficial than the gene-based approach in identifying common biological features in HCC recurrence. For this purpose, we developed a pathway-based PCA algorithm. Previously, PCA using a subset of genes was reported to accurately predict clinical outcomes of patients [14]. Here, we used an optimal component, rather than the first or second principal component. Despite the rarity of common genes between the two HBV-positive HCC datasets, a large proportion (71.9%) of the significant pathways overlapped. Interestingly, 16 pathways (28.1%) were common among our HBV-positive HCC group and the two public HBV and HCV-positive HCC datasets, as shown in Figure 4D (see also Additional file 3: Table S3).

Our cohort exhibited a biphasic pattern in which the majority of recurrence occurred within the first two years, similar to the public HBV-HCC cohort [3]. Considering that recurrence is critically linked to survival in HCC, understanding the pathways associated with early recurrence is crucial for effective clinical management of the disease. Our pathway-based approach using optimal principal components was associated with early recurrence occurring within two years, with high sensitivity and specificity in ROC curve analysis. Interestingly, patients with poor prognosis could be distinguished among those with small tumors using the present approach. The finding that recurrence-associated pathways may be an independent variable for HCC recurrence along with tumor number, tumor size and Edmondson’s grade supports the clinical utility of our method.

Although the 16 pathways were common in both HBV- and HCV-HCC datasets, their clinical advantage in patient classification was not clear. Since these common pathways were not optimal, we obtained reduced performance in classification of patients. In addition to its clinical value, the overlap of significant pathways between HBV- and HCV-positive datasets further suggests that recurrence of HBV- and HCV-positive HCCs is commonly affected by dysfunction of common biological pathways (including hemostasis, platelet activation, transmembrane transporter, actin cytoskeleton, axon guidance, immune signaling, MAPK signaling, ErbB signaling, and GPCR signaling). Notably, these pathways have already been reported in association with development or prognosis in diverse types of human cancers, including HCC. For example, hemostasis and platelet-related pathways are critical in cancer development [21,22]. Signaling pathways, such as MAPK and ErbB, and immune-related pathways are involved in prognosis and tumorigenesis of HCC [23-26]. G-protein pathways, including GPCR signaling, are de-regulated in diverse types of cancers and can thus be effectively used as prognostic markers [27]. Moreover, enrichment of immune response pathways (25%, 16 out of 64 recurrence-associated pathways in Additional file 3: Table S3) supports a close association of the high-risk subgroup with lobular activity, a clinical parameter for inflammation (Table 1). Lobular activity status is related to not only these recurrence-associated pathways, but also a subset of recurrence-associated genes, as shown in Additional file 9: Figure S6. Under the condition where 206 genes were selected as associated with lobular activity (p < 0.001, t-test), 23 (p < 0.01, log-rank test) were related to recurrence (p < 0.0001, chi-square test). These gene sets exhibited differential expression profiles according to differences in lobular activity. Other diverse pathways, such as lysosome, endocytosis, and hemostasis, were additionally associated with recurrence. Although the functional involvement of these pathways in human cancer has not been fully elucidated, studies have shown that the lysosomal pathway is a novel target of cell death in cancer [28]. Moreover, lysosomal proteins, such as lysosome-associated membrane protein-1 [29] and lysosomal cysteine proteases [30], are associated with tumor development and progression. The clathrin-mediated endocytosis pathway connected with the cytoskeleton has been shown to regulate functional changes in HCC [31]. This association of diverse functional pathways clearly indicates that recurrence is a complex process involving changes in various biological functions.

In contrast to the common pathways discussed above, tight junction, focal adhesion and integrin pathways were more significantly associated with HBV-positive HCC. These membrane mobility and intercellular interaction pathways are involved in HCC invasion, metastasis and angiogenesis [32-34]. Interestingly, sphingolipid metabolism was mainly associated with recurrence of HCV-positive HCC. Accumulating evidence suggests that sphingolipid regulates cell death in liver diseases, including HCC [35,36].

Previously, we reported functional differences and similarities in pathways implicated in the development of HCV- and/or HBV-positive HCC [12,37]. By comparing these tumorigenic pathways with recurrence-associated pathways, we obtained those commonly involved in both processes in HBV-positive and HCV-positive HCC. In the HBV-positive HCC dataset, 26 out of 64 recurrence-associated pathways (40.6%) were associated with tumorigenesis, while 12 out of 32 recurrence-associated pathways (37.5%) were involved in tumorigenesis in the public HCV-HCC dataset (Additional file 10: Figure S7). Involvement of a relatively large proportion of recurrence pathways in tumorigenesis signifies that the biological processes associated with hepatocarcinogenesis also determine the prognostic process. Moreover, a number of pathways, including axon guidance, lysosome, actin cytoskeleton, and GPCR signaling, were shared between the HBV-HCC and HCV-HCC datasets (Additional file 11: Figure S8), highlighting the possibility that a common process affects tumor development to prognosis, irrespective of the viral origin of HCC.

We further validated our pathway-based classification of HCC patients with those published employing prognostic genes for HCC. Survival-associated genes identified by Lee *et al*. [2] and Hoshida *et al*. [8], as well as recurrence-associated genes from the public HBV- and HCV-HCC datasets, led to grouping of patients into subclasses significantly associated with those obtained with the pathway-based approach using 64 pathways (Additional file 12: Figure S9). Among the patients classified as high-risk based on our 64 recurrence-associated pathways, a significant number (p < 0.05) belonged to the high-risk subclasses identified based on each of the four different survival or recurrence gene sets. This comparative result further confirmed that our pathway-based classification of HCC prognosis is compatible with prior classification systems using survival- and recurrence-associated genes.

## Conclusions

Our pathway-based approach using optimal PCA was more advantageous than the gene-based approach in several aspects, and revealed common prognostic pathways between HCC datasets of different patient cohorts, despite the rarity of common recurrence-associated genes. Identification of these shared pathways allowed the evaluation of functional similarities and differences between HBV- and HCV-positive HCCs. Further studies are essential to validate the specific functions of these pathways in addition with HCC recurrence.

## Methods

### HCC dataset

The HBV-positive HCC dataset (HBV-HCC) was composed of 142 HCC tissues from patients subjected to surgical resection at Korea Cancer Center Hospital and Seoul National University Hospital at Korea from March 1992 to July 2004. The tissue specimens were immediately collected after resection, and stored in liquid nitrogen until RNA extraction for microarray experiment. The present retrospective study was approved by the Institutional Review Boards of Seoul National University Hospital and Korea Cancer Center Hospital. Written informed consent was obtained from the patients of Seoul National University Hospital or waived by the Institutional Review Board of Korea Cancer Center Hospital. Prior to analysis, patient information was anonymized and de-identified. Clinicopathologic data were obtained retrospectively, including platelet, bilirubin, alanine transaminase, aspartate aminotransferase, α-fetoprotein, Child-Pugh score, tumor size, tumor number, tumor grade, vein invasion, lobular activity, capsule formation, resection margin, and cirrhosis.

### Microarray experiment

Total RNA from HCC tissues was extracted using the Qiagen RNeasy Mini Kit (Qiagen, Valencia, CA), in accordance with the manufacturer’s instructions. The quantity and quality of purified RNA were measured using an Agilent 2100 Bioanalyzer (Agilent Technologies, Santa Clara, CA), and only samples with RNA integrity number (RIN) greater than 7.0 were included in the microarray analysis [38,39]. Total RNA isolated from frozen tissues was reverse-transcribed to cDNA, labeled with fluorescent dye (Cy5 for HCC and Cy3 for reference normal liver control) and hybridized to microarray using a 3-DNA array detection system (Genisphere, Hatfield, USA). Equal mixtures of total RNA from five metastatic liver cancers with no evidence of cirrhosis, which originated from other organs, were used as a reference control. Approximately 25,000 cDNAs (representing ~11,000 genes) were printed onto glass microscope slides. Hybridized microarrays were scanned using a ScanArray (Perkin-Elmer, Boston, USA). Primary signal intensities were obtained using ImaGene 6.0 software (Bio-discovery, Marina del Rey, USA). The distribution of signal intensities of fluorescent probes was assessed using MA and QQ plots to exclude inadequate samples for analysis (Additional file 13: Figure S10). Only spots for which signal intensities were greater than 1.4-fold, compared to those of the local background, were normalized using the lowess and quantile method to eliminate intensity bias [40,41]. After averaging the ratios of multiple probes per single gene, 9,357 genes were included in the present study.

### Public microarray datasets

Two publicly available microarray datasets were utilized for comparison. The first dataset (Public HBV-HCC), archived in the Gene Expression Omnibus with the accession number GSE14520, is composed of 236 HBV-positive HCC specimens [3], and provides information on gene expression based on a single-channel array platform (Affymetrix GeneChip HG-U133A). We normalized the probe intensities of each array using the quantile method [41]. After averaging multiple probes per single gene, 13,345 genes were ultimately subjected to analysis. The second set (Public HCV-HCC) is composed of 43 HCV-positive specimens (GSE17856) [4]. Gene expression of this dataset is based on oligo-microarrays (Agilent-014850 Whole Human Genome Microarray) in which Cy5 and Cy3 fluorescent dyes were used to label RNAs from tissue samples and reference control, respectively. We normalized logarithm values of the probe intensity ratio (Cy5/Cy3) using the quantile method [41]. Multiple probes per single gene were averaged to produce a final set of 14,411 genes. Clinical comparisons of the three datasets are presented in Additional file 2: Table S1.

### Identification of recurrence-related genes

Genes associated with recurrence were selected using BRB ArrayTools (version 3.6, http://linus.nci.nih.gov/BRB-ArrayTools.html) in which expression values are correlated with recurrence time using the Cox Proportional Hazards Model. The log-rank *p*-values for individual genes were tested one at a time for the hypothesis that recurrence time is independent of the expression level for that gene. The *p*-values were applied to construct gene lists using multivariate permutation tests for controlling the number or proportion of false discoveries with 10,000 iterations.

### Pathway-based supervised Principal Component Analysis

Initially, samples were randomly split into two groups: training (60%) and test sets (40%). PCA analysis was performed in the training set with genes belonging to each pathway. First, in each pathway, we selected a single optimal principal component most significantly associated with recurrence in Cox proportional hazards regression. The optimal principal component was subsequently applied to the test set to obtain sample scores. In weighted PCA, the principal components responsible for over 80% variance in the training set were selected and linearly added to generate the sample score using regression coefficients of individual components as weight. This procedure was repeated 1,000 times to facilitate maximum extraction of information from all samples. Next, the median value of statistics over the entire permutation process was computed on each pathway. The statistical significance of the test validation outcomes was computed by comparing with results from randomly selected pathways with 1,000 iteration times. All procedures were performed in R (v2.14.0). The R source code for the program is available on request.

### Determination of the threshold p-value

Genes were ranked and ordered according to log-rank *p-*values on Cox proportional hazards regression. We applied permutation-based (n = 1,000) pathway analysis, as described above, starting from genes with the lowest univariate *p-*value and gradually increasing the number of genes, and measured the average number of pathways below the log-rank *p-*value of 0.01 in the test set. The threshold was taken as the *p-*value with the maximum number of significant pathways in the test set.

### Pathway information

Manually curated pathways were obtained from the Molecular Signatures Database (MSigDB, http://www.broadinstitute.org/gsea) [10] including 186 pathways from Kyoto Encyclopedia of Genes and Genomes [42], 217 from BioCarta (http://www.biocarta.com/genes/allPathways.asp) and 430 from Reactome [43]. Among a total of 833 pathways, only those containing at least 10% of microarray genes in each pathway were used in the experiment.

### Pathway cluster

For pathway-based clustering of samples, we constructed a matrix composed of optimal principal component scores for each sample and pathway. The matrix was hierarchically clustered based on similarities of the principal components to classify the samples [44]. For robustness of the resultant subclasses, cross-validation using the leave-one-out method was implemented in BRB ArrayTools using six different validation algorithms, including the compound covariate, diagonal linear discriminant, 1-nearest neighbor, 3-nearest neighbor, nearest centroid and support vector machine. The statistical significance of misclassification error was determined using random permutation of samples (N = 1,000). The differences in recurrence rates between subclasses were estimated using the Kaplan-Meier method with log-rank test. ROC curve for recurrence assessment was measured from the compound covariate algorithm for subgroup classification using the survival ROC R package [45].

### Other pathway-based analyses

For a pathway composed of N genes, LS statistics is used to measure the average value of negative natural logarithms of single gene *p-*values. KS statistics allow calculation of the maximum difference between *i*/N and *i*^th^ smallest *p-*value. The significance of LS and KS was evaluated by computing the distribution of these statistics in a random sampling of 1,000 times [46]. ROC-AUC for each pathway was evaluated from the values of principal components with the survival ROC R package.

## Availability of supporting data

Our HBV-HCC microarray dataset is available online at the Gene Expression Omnibus (http://www.ncbi.nlm.nih.gov/geo) under the ID number GSE55039.

## Additional files

Additional file 1: Table S1. Top 20 recurrence-associated genes in our HBV-HCC dataset. Additional file 2: Table S2. Clinicopathological information on three HCC datasets. Additional file 3: Table S3. Pathways associated with recurrence in each HCC dataset. Additional file 4: Figure S1. Comparison of one-optimal PCA and weighted PCA methods. Log-rank *p-*values of 882 pathways calculated with the two methods were compared. One-optimal PCA used one principal component optimally associated with recurrence while weighted PCA used multiple principal component sex plaining over 80% variance in each dataset. Additional file 5: Figure S2. Association of the axon guidance pathway with recurrence of HCC. Kaplan-Meier plot sand receiver operating characteristic (ROC) curve with the axon guidance pathway in HBV-HCC, public HBV-HCC and public HCV-HCC. For the Kaplan-Meier plot, samples were classified in to two subgroups based on the median value of principal components of the axon guidance pathway. Additional file 6: Figure S3. Cluster analysis of recurrence-related pathways. (A) Dendrogram of clustering pattern measured from the matrix of principal components of 90 recurrence-associated pathways (*p-*value < 0.01) from public HBV-HCC. Samples were classified into two subgroups: low-risk and high-risk based on recurrence out come. Columns represent individual samples, and rows pathways. Red and green colors reflect high and low levels of optimal principal component scores, respectively, as indicated by scale bars. (B) Kaplan-Meier plots for recurrence rates of the low-and high-risk subgroups. (C) Cross-validation of the low-and high-risk subgroups using six different algorithms: compound covariate (CC), diagonal line ardiscriminant (DLD), 1-nearest neighbor (1-NN), 3-nearest neighbor (3-NN), nearest centroid (NC) and support vector machine (SVM). (D) ROC curve computed with CC validation algorithm for subgroup classification. Additional file 7: Figure S4. Cluster analysis of recurrence-related pathways. (A) Dendrogram of clustering pattern measured from the matrix of principal components of 32 recurrence-associated pathways (*p-*value < 0.01) from public HCV-HCC. Samples were classified into two subgroups: low-risk and high-risk based on recurrence out come. Columns represent individual samples, and rows pathways. Red and green colors reflect high and low levels of optimal principal component scores, respectively, as indicated by scale bars. (B) Kaplan-Meier plots for recurrence rates of the low-and high-risk subgroups. (C) Cross-validation of the low-and high-risk subgroups using six different algorithms: compound covariate (CC), diagonal line ardiscriminant (DLD), 1-nearest neighbor (1-NN), 3-nearest neighbor (3-NN), nearest centroid (NC) and support vectormachine (SVM). (D) ROC curve computed with CC validation algorithm for subgroup classification. Additional file 8: Figure S5. Stratification of small tumor-size patients by recurrence-associated pathways. (A) Kaplan-Meier plot for recurrence rates in two subgroups of patients based on tumorsize (<and > 5 cm) in public HBV-HCC dataset. (B) Application of subgroup information determined from recurrence-associated pathways to small tumor-size patients (<5 cm) led to further stratification into two subgroups with different recurrence rates. Additional file 9: Figure S6. Genes associated with lobular activity. A total of 206 genes were selected as differentially expressed (p < 0.001 with t-test) according to status of lobular activity. Among these genes, 23 were significantly associated with recurrence using the log-rank test (p < 0.01). Additional file 10: Figure S7. Comparison of recurrence-associated and tumorigenic pathways. Recurrence-associated path ways identified in the present study were compared with those associated with tumorigenesis in HCC obtained from our prior analysis of the same public HBV and HCC datasets [12]. In total, (A) 64 recurrence-associated pathways from our HBV-HCC dataset (log-rank p-value < 0.01 in the testset) were compared with 84 tumorigenic pathways from another HBV-positive HCC dataset (error rate < 0.1), and (B) 32 recurrence-associated pathways from public HCV-HCC (log-rank p-value < 0.01 in the testset) compared with 138 tumorigenic pathways from the same dataset (error rate < 0.1). Additional file 11: Figure S8. Distribution of recurrence-associated and tumorigenic pathways. Additional file 12: Figure S9. Comparison of patient classification. With previously reported prognosis-associated genes (Lee *et al*. [2], Hoshida *et al*. [8], public HBV-HCC [3] and public HCV-HCC u datasets), patient subclasses were determined using hierarchical clustering, and compared with subgroups obtained from our pathway-based method. Blue and red colors represent low-and high-risk subgroups, respectively, while white (public HBV-HCC) signifies the intermediate subgroup. Statistical significance was measured using Fisher’s exact test in comparison with pathway-based low-and high-risk subclasses. Additional file 13: Figure S10. MA and QQ plots for assessing sample quality before normalization. The distribution of signal intensities of fluorescent probes was examined using MA and QQ plots. Older and newer samples indicate those collected before 1995 and after 2000, respectively. For QQ plot, we used the newest sample, S174, as a common reference.

## Abbreviations

HCC: Hepatocellular carcinoma
HBV: Hepatitis B virus
HCV: Hepatitis C virus
PCA: Principle component analysis
AUC: Area under curve
ROC: Receiver operating characteristics
