# Supplementary material for: Recurrence-associated pathways in hepatitis B virus-positive hepatocellular carcinoma
Source: BMC Genomics. 2015 Apr 10;16(1):279. doi: 10.1186/s12864-015-1472-x (PMC4448317; doi:10.1186/s12864-015-1472-x)
Supplement: Additional file 1: Table S1. — Top 20 recurrence-associated genes in our HBV-HCC dataset. [file 12864_2015_1472_MOESM1_ESM.pdf]

Table S1. Top 20 recurrence-associated genes in our HBV-HCC dataset

Symbol	Log-rank *p*-value	FDR[1]	Permutation p-value[2]	Hazard ratio (95% CI)	Function	References[3]
KNG1	5.30E-05	0.194	< 1E-07	0.579 (0.444 0.754)	Blood coagulation	1
TM4SF1	6.69E-05	0.194	2.00E-04	3.100 (1.777 5.405)	Angiogenesis	2,3
LRRC31	8.25E-05	0.194	5.00E-04	0.389 (0.243 0.622)		
POLR3GL	9.89E-05	0.194	1.00E-04	0.333 (0.191 0.579)	Polymerase	4
ZNF10	0.000131	0.194	0.0013	0.501 (0.351 0.713)	Transcription	5
DARS	0.000135	0.194	< 1E-07	4.563 (2.093 9.950)	Translation	6
NUAK1	0.000169	0.194	2.00E-04	3.189 (1.743 5.835)	Signaling	7,8,9
CFL1	0.000188	0.194	3.00E-04	6.145 (2.370 15.940)	Invasion	10,11
EEPD1	0.000211	0.194	4.00E-04	2.768 (1.615 4.742)	DNA repair	12
CCDC112	0.000220	0.194	1.00E-04	7.042 (2.500 19.830)		
TRAFD1	0.000228	0.194	0.0033	1.837 (1.330 2.539)	Signaling	13
FCGR3B	0.000267	0.208	3.00E-04	2.117 (1.414 3.169)	Immune	14,15
SCN9A	0.000330	0.236	1.00E-04	4.166 (1.912 9.080)	Neuronal disease	16
C10orf10	0.000352	0.236	4.00E-04	1.877 (1.329 2.652)	Signaling	17,18
S100A9	0.000409	0.249	5.00E-04	1.452 (1.181 1.786)	Cancer	19,20
TMEM141	0.000425	0.249	5.00E-04	3.677 (1.782 7.587)		
LRRC20	0.000462	0.254	3.00E-04	0.294 (0.148 0.583)	Immune	21
B3GNT2	0.000510	0.257	3.00E-04	0.411 (0.249 0.678)	Metabolism Immune	22
C1D	0.000521	0.257	7.00E-04	4.022 (1.832 8.829)	Apoptosis	23
YTHDF2	0.000569	0.265	5.00E-04	5.174 (2.032 13.180)	Cancer	24,25
SAPS3	0.000595	0.265	0.0012	0.239		
PSMA5	0.000699	0.281	6 E-04	2.302		
MAP7D3	0.000704	0.281	0.0012	6.264		
ARFGAP3	0.000746	0.281	8 E-04	5.203		
LRRFIP1	0.000750	0.281	0.0011	7.959		
CPNE3	0.000809	0.283	7 E-04	4.229		
EXTL3	0.000836	0.283	0.0012	0.265		
ST8SIA4	0.000848	0.283	8 E-04	0.401		
SERPINB1	0.000876	0.283	0.0013	3.934		
ARPC1B	0.000960	0.3	5 E-04	2.266		
CEP350	0.00114	0.346	0.0012	0.181		
EIF3M	0.00118	0.346	0.0013	4.562		
CALU	0.00124	0.346	0.0014	4.674		
CYP11A1	0.00125	0.346	0.0014	0.241		
TMPO	0.00133	0.349	0.0016	0.359		

GPR126	0.00134	0.349	0.0014	4.343
ZC3H15	0.00156	0.357	0.0021	3.825
CREM	0.00159	0.357	0.0012	2.442
WDR90	0.00165	0.357	0.0019	2.432
LOC643684	0.00170	0.357	0.0016	0.357
HIAT1	0.00180	0.357	0.0018	5.953
GPR135	0.00202	0.357	0.0062	0.257
EML3	0.00208	0.357	0.0022	0.354
ALS2CL	0.00210	0.357	0.0022	0.405
NOC2L	0.00217	0.357	0.0021	5.558
WHSC1L1	0.00218	0.357	0.0025	2.329
MAN2B1	0.00218	0.357	0.0034	2.615
ZNF609	0.00225	0.357	0.0025	0.285
SLC16A1	0.00228	0.357	0.0032	2.074
FHOD3	0.00229	0.357	0.0018	3.764
FN1	0.00231	0.357	0.0018	2.224
INTS5	0.00238	0.357	0.0026	0.31
SDAD1	0.00245	0.357	0.0019	3.704
KIAA0430	0.00247	0.357	0.0041	1.921
XPA	0.00255	0.357	0.0025	5.208
APOA1	0.00257	0.357	0.0019	0.755
FUT1	0.00264	0.357	0.003	0.378
CDC27	0.00274	0.357	0.0026	0.141
FAM125B	0.00275	0.357	0.0026	4.562
MST1R	0.00276	0.357	0.0033	0.155
SAMHD1	0.00278	0.357	0.0036	2.432
DDX26B	0.00279	0.357	0.0031	2.876
APOA2	0.00280	0.357	0.0033	0.755
EP400NL	0.00284	0.357	0.0025	0.24
CORO1C	0.00284	0.357	0.0022	3.068
CLDN15	0.00287	0.357	0.0026	0.319
KRT19	0.00287	0.357	0.003	2.612
JAGN1	0.00293	0.357	0.0033	2.738
EVC	0.00293	0.357	0.003	2.616
ATP6V0C	0.00303	0.357	0.0029	4.572
PDGFC	0.00304	0.357	0.0038	1.949
TMEM166	0.00307	0.357	0.0042	2.38
C11orf41	0.00309	0.357	0.0034	1.673
SRGAP3	0.00311	0.357	0.0033	3.204
CSNK1A1	0.00313	0.357	0.0031	0.296
TLK2	0.00315	0.357	0.0047	2.053
HOXA13	0.00315	0.357	0.0032	2.701
LY6E	0.00317	0.357	0.0038	1.912
ZNF205	0.00317	0.357	0.0028	0.314
GMFB	0.00318	0.357	0.0047	0.358
JDP2	0.00318	0.357	0.0031	0.315
SLC6A1	0.00318	0.357	0.0028	0.521
GABRA1	0.00322	0.357	0.0048	0.568

FXN	0.00323	0.357	0.0028	3.536
USF1	0.00327	0.357	0.0022	0.352
MAP2K3	0.00328	0.357	0.0027	3.575
ZNF180	0.00341	0.357	0.004	0.225
LRPPRC	0.00343	0.357	0.003	2.569
ANXA5	0.00345	0.357	0.0034	2.3
C11orf31	0.00354	0.357	0.0029	3.398
AACS	0.00358	0.357	0.0044	5.457
RAB43	0.00359	0.357	0.0039	0.448
MAST3	0.00362	0.357	0.0028	0.179
GPR175	0.00367	0.357	0.0041	0.278
ZNF433	0.00377	0.357	0.0043	0.467
IL12A	0.00383	0.357	0.0036	0.446
CHD1	0.00383	0.357	0.004	0.242
CNOT6	0.00385	0.357	0.0048	0.144
TCEAL8	0.00387	0.357	0.0034	0.373
PTP4A2	0.00387	0.357	0.0042	4.359
EPDR1	0.00396	0.357	0.0032	2.08
COL17A1	0.00396	0.357	0.0042	0.373
PCBP2	0.00397	0.357	0.005	2.564
PDE1A	0.00397	0.357	0.0048	2.013
GRPEL2	0.00400	0.357	0.0042	2.64
UTP3	0.00408	0.361	0.0077	3.815
MRPS2	0.00415	0.361	0.0032	4.386
CRKL	0.00418	0.361	0.005	0.219
FAM84A	0.00420	0.361	0.0048	5.384
EIF1	0.00433	0.369	0.0029	2.702
FAM118B	0.00440	0.371	0.0072	0.582
KLF16	0.00456	0.374	0.0051	2
VASP	0.00458	0.374	0.0066	5.847
ALS2	0.00460	0.374	0.0044	0.265
RGS10	0.00469	0.374	0.0047	0.356
RHOBTB1	0.00470	0.374	0.005	2.365
SILV	0.00471	0.374	0.0041	0.264
ACOX1	0.00471	0.374	0.0053	4.503
C20orf59	0.00478	0.374	0.0045	0.203
CCL24	0.00480	0.374	0.0053	0.481
VSIG4	0.00484	0.374	0.0064	1.799
PER1	0.00490	0.374	0.0062	3.796
SHPRH	0.00492	0.374	0.0059	0.304
SLC35F2	0.00496	0.375	0.005	0.216
KIAA1949	0.00501	0.375	0.0052	2.474
ESRRA	0.00507	0.377	0.0059	0.116
SOD2	0.00525	0.387	0.006	1.774
FAM122B	0.00531	0.389	0.0063	0.232
SLC41A1	0.00543	0.389	0.0054	1.616
C18orf55	0.00549	0.389	0.0063	0.316
CTSL1	0.00552	0.389	0.0066	1.809

RACGAP1	0.00553	0.389	0.0061	3.085
C11orf77	0.00554	0.389	0.0075	0.349
LOC388210	0.00556	0.389	0.0051	0.253
PSMG3	0.00573	0.39	0.006	1.734
FMNL1	0.00573	0.39	0.0062	0.344
SYNPO	0.00580	0.39	0.0054	0.382
PNPLA6	0.00584	0.39	0.006	0.171
ASPH	0.00586	0.39	0.0068	3.598
C11orf82	0.00593	0.39	0.0059	0.52
NFKBIB	0.00599	0.39	0.006	0.463
GTF3A	0.00600	0.39	0.0049	2.987
LOC100131861	0.00604	0.39	0.0069	0.337
UBTD2	0.00605	0.39	0.006	0.252
FCF1	0.00612	0.39	0.008	0.322
CLSPN	0.00615	0.39	0.0052	0.265
JUN	0.00616	0.39	0.0075	2.766
CCDC14	0.00616	0.39	0.0054	0.327
TBC1D13	0.00626	0.392	0.007	0.308
PFDN5	0.00630	0.392	0.0064	2.762
PCSK5	0.00634	0.392	0.007	0.369
PPBP	0.00641	0.392	0.0066	3.346
CCT2	0.00644	0.392	0.0088	2.13
KIAA0196	0.00644	0.392	0.007	2.474
SPI1	0.00653	0.392	0.0062	0.452
ZSCAN16	0.00660	0.392	0.0082	0.468
HOMER1	0.00668	0.392	0.0069	2.31
GSTA1	0.00670	0.392	0.0065	0.732
CEBPG	0.00670	0.392	0.0066	0.169
CASC3	0.00671	0.392	0.0072	0.317
ANP32A	0.00677	0.392	0.0072	0.466
PAQR8	0.00683	0.392	0.0069	0.247
CISD3	0.00684	0.392	0.006	3.885
BOP1	0.00692	0.392	0.0072	2.004
ZNF638	0.00698	0.392	0.0074	0.194
TRIT1	0.00705	0.392	0.008	3.344
TNFAIP3	0.00708	0.392	0.0065	1.636
HMGB1	0.00708	0.392	0.0084	0.117
TUFM	0.00708	0.392	0.007	2.226
SULT1A1	0.00717	0.393	0.0079	1.75
PTPRG	0.00718	0.393	0.0078	2.906
DDX21	0.00732	0.397	0.0065	2.77
SYT7	0.00733	0.397	0.0075	0.367
LITAF	0.00752	0.405	0.0064	3.716
LBP	0.00757	0.405	0.0064	1.633
C14orf131	0.00774	0.411	0.0093	0.309
HIF1A	0.00784	0.411	0.009	1.721
NEIL3	0.00796	0.411	0.0096	2.926
MCCC1	0.00799	0.411	0.008	0.191

ASB3	0.00801	0.411	0.0105	0.569
FLJ11151	0.00803	0.411	0.0083	2.295
IFIT2	0.00805	0.411	0.0086	0.374
ABCD3	0.00807	0.411	0.009	3.193
APLP2	0.00808	0.411	0.0077	2.617
NPAL2	0.00819	0.414	0.0088	0.236
POLR3G	0.00827	0.416	0.0111	0.54
CAP1	0.00841	0.421	0.0082	0.407
CXCL5	0.00846	0.421	0.0102	3.774
RNF141	0.00859	0.423	0.0084	3.324
ZNF345	0.00863	0.423	0.0113	0.438
AK3	0.00864	0.423	0.0083	2.574
LOC152742	0.00883	0.429	0.0096	0.219
PA2G4	0.00885	0.429	0.0083	4.181
KCMF1	0.00890	0.43	0.0088	2.791
FOXP3	0.00895	0.43	0.0103	0.341
SMN2	0.00902	0.431	0.0116	0.301
PCTK2	0.00910	0.432	0.0074	0.236
SERBP1	0.00922	0.436	0.011	4.659
CA4	0.00935	0.437	0.0134	2.558
CCDC23	0.00938	0.437	0.0095	2.866
LRP8	0.00938	0.437	0.0118	0.487
DYRK2	0.00949	0.437	0.0105	0.229
FOS	0.00956	0.437	0.0103	1.622
CXCL1	0.00959	0.437	0.0088	1.677
RAB20	0.00961	0.437	0.0103	2.506
TMEM194	0.00962	0.437	0.0107	0.485
KIAA0895	0.00973	0.44	0.0101	0.317
DDX19B	0.00988	0.445	0.0113	2.066
ABHD5	0.00994	0.445	0.0105	2.483

[1]False discovery rate (FDR) was calculated with a univariate permutation test in BRB Array Tools.

[2]The number of random permutations was set to 10,000.

[3]Supplementray references for functions of top 20 genes are listed at the end of Supplementary material.

Supplementary References

1. Morange PE, Oudot-Mellakh T, Cohen W, Germain M, Saut N, et al. (2011) KNG1 Ile581Thr and susceptibility to venous thrombosis. Blood 117: 3692-3964.

2. Allioli N, Vincent S, Vlaeminck-Guillem V, Decaussin-Petrucci M, Ragage F, et al. (2011) TM4SF1, a novel primary androgen receptor target gene over-expressed in human prostate cancer and involved in cell migration. Prostate 71: 1239-1250.

3. Zukauskas A, Merley A, Li D, Ang LH, Sciuto TE, et al. (2011) TM4SF1: a tetraspanin-like protein necessary for nanopodia formation and endothelial cell migration. Angiogenesis 14: 345-354.

4. Haurie V, Durrieu-Gaillard S, Dumay-Odelot H, Da Silva D, Rey C, et al. (2010) Two isoforms of human RNA polymerase III with specific functions in cell growth and transformation. Proc Natl Acad Sci USA 107: 4176-4181.

5. Lorenz P, Koczan D, Thiesen HJ (2001) Transcriptional repression mediated by the KRAB domain of the human C2H2 zinc finger protein Kox1/ZNF10 does not require histone deacetylation. Biol Chem 382: 637-644.

6. Cheong HK, Park JY, Kim EH, Lee C, Kim S, et al. (2003) Structure of the N-terminal extension of human aspartyl-tRNA synthetase: implications for its biological function. Int J Biochem Cell Biol 35: 1548-1557.

7. Hou X, Liu JE, Liu W, Liu CY, Liu ZY, et al. (2011) A new role of NUAK1: directly phosphorylating p53 and regulating cell proliferation. Oncogene 30: 2933-2942.

8. Suzuki A, Lu J, Kusakai G, Kishimoto A, Ogura T, et al. (2004) ARK5 is a tumor invasion-associated factor downstream of Akt signaling. Mol Cell Biol 24: 3526-3535.

9. Kusakai G, Suzuki A, Ogura T, Miyamoto S, Ochiai A, et al. (2004) ARK5 expression in colorectal cancer and its implications for tumor progression. Am J Pathol 164: 987-995.

10. Castro MA, Dal-Pizzol F, Zdanov S, Soares M, Müller CB, et al. (2010) CFL1 expression levels as a prognostic and drug resistance marker in nonsmall cell lung cancer. Cancer 116: 3645-3655.

11. Peng XC, Gong FM, Zhao YW, Zhou LX, Xie YW, et al. (2011) Comparative proteomic approach identifies PKM2 and cofilin-1 as potential diagnostic, prognostic and therapeutic targets for pulmonary adenocarcinoma. PLoS One 6: e27309.

12. Gene Ontology [http://www.geneontology.org]

13. Sanada T, Takaesu G, Mashima R, Yoshida R, Kobayashi T, et al. (2008) FLN29 deficiency reveals its negative regulatory role in the Toll-like receptor (TLR) and retinoic acid-inducible gene I (RIG-I)-like helicase signaling pathway. J Biol Chem 283: 33858-33864.

14. McKinney C, Merriman TR (2012) Meta-analysis confirms a role for deletion in FCGR3B in autoimmune phenotypes. Hum Mol Genet 21: 2370-2376.

15. Morris DL, Roberts AL, Witherden AS, Tarzi R, Barros P, et al. (2010) Evidence for both copy number and allelic (NA1/NA2) risk at the FCGR3B locus in systemic lupus erythematosus. Eur J Hum Genet 18: 1027-1031.

16. Cox JJ, Reimann F, Nicholas AK, Thornton G, Roberts E, et al. (2006) An SCN9A channelopathy causes congenital inability to experience pain. Nature 444: 894-898.

17. Kuroda Y, Kuriyama H, Kihara S, Kishida K, Maeda N, et al. (2010) Insulin-mediated regulation of decidual protein induced by progesterone (DEPP) in adipose tissue and liver. Horm Metab Res 42: 173-177.

18. Watanabe H, Nonoguchi K, Sakurai T, Masuda T, Itoh K, et al. (2005) A novel protein Depp, which is induced by progesterone in human endometrial stromal cells activates Elk-1 transcription factor. Mol Hum Reprod 11: 471-476.

19. Németh J, Stein I, Haag D, Riehl A, Longerich T, et al. (2009) S100A8 and S100A9 are novel nuclear factor kappa B target genes during malignant progression of murine and human liver carcinogenesis. Hepatology 50: 1251-1262.

20. Källberg E, Vogl T, Liberg D, Olsson A, Björk P, et al. (2012) S100A9 interaction with TLR4 promotes tumor growth. PLoS One 7: e34207.

21. Kariuki SN, Franek BS, Kumar AA, Arrington J, Mikolaitis RA, et al. (2010) Trait-stratified genome-wide association study identifies novel and diverse genetic associations with serologic and cytokine phenotypes in systemic lupus erythematosus. Arthritis Res Ther 12: R151.

22. Togayachi A, Kozono Y, Kuno A, Ohkura T, Sato T, et al. (2010) Beta3GnT2 (B3GNT2), a major polylactosamine synthase: analysis of B3GNT2-deficient mice. Methods Enzymol 479: 185-204.

23. Li G, Liu J, Abu-Asab M, Masabumi S, Maru Y (2010) XPB induces C1D expression to counteract UV-induced apoptosis. Mol Cancer Res 8: 885-895.

24. Heiliger KJ, Hess J, Vitagliano D, Salerno P, Braselmann H, et al. (2012) Novel candidate genes of thyroid tumourigenesis identified in Trk-T1 transgenic mice. Endocr Relat

Cancer 19: 409-421

25. Nguyen TT, Ma LN, Slovak ML, Bangs CD, Cherry AM, et al. (2006) Identification of novel Runx1 (AML1) translocation partner genes SH3D19, YTHDf2, and ZNF687 in acute myeloid leukemia. Genes Chromosomes Cancer 45: 918-932.
