# Supplementary material for: Recurrence-associated pathways in hepatitis B virus-positive hepatocellular carcinoma
Source: BMC Genomics. 2015 Apr 10;16(1):279. doi: 10.1186/s12864-015-1472-x (PMC4448317; doi:10.1186/s12864-015-1472-x)
Supplement: Additional file 2: Table S2. — Clinicopathological information on three HCC datasets. [file 12864_2015_1472_MOESM2_ESM.pdf]

Table S2. Clinicopathological information on three HCC datasets

Clinical variables[1]	HBV-HCC	Public HBV-HCC[2]	Public HCV-HCC[2]	P-value[3]
Age (year)	51.8±9.5	50.7±10.8	66.5±7.9	
Male number	117 (82.4%)	205 (86.9%)	32 (74.4%)	
Viral infection				
HCV infection	0 (0%)	0 (0%)	43 (100%)	
HBV infection	142 (100%)	236 (100%)	0 (0%)	
Total bilirubin (mg/dl)	1.05±1.28	-	0.80±0.35	
Alanine aminotransferase (IU/l)	44.8±26.0	>50 IU/L<br>No. 99 (41.9%)	51.7±33.1	
Alpha-fetoprotein (ng/ml)	>300 ng/ml<br>No. 48 (34.0%)	>300 ng/ml<br>No. 107 (46.1%)	345±962	
Tumor diameter (cm)	5.85±3.74	>5cm<br>No. 84 (35.7%)	2.95±1.37	
Number of tumors	1.14±0.35	Single nodule<br>No. 184 (78.0%)	1.30±0.51	
Tumor differentiation				0.112
Well differentiated	11 (8.02%)	-	6 (14.0%)	
Moderately differentiated	79 (57.7%)	-	29 (67.4%)	
Poorly differentiated	47 (34.3%)	-	8 (18.6%)	
Tumor stage				1.064×10^{-5}
I	32 (23.5%)	92 (42.0%)	8 (18.6%)	
II	57 (41.9%)	77 (35.2%)	24 (55.8%)	
III	37 (27.2%)	50 (22.8%)	10 (23.3%)	
IV	10 (7.35%)	0 (0%)	1 (2.3%)	
Fibrosis stage				0.566
0	8 (2.1%)	-	0 (0%)	
1	11 (10.5%)	-	3 (7.0%)	
2	21 (13.7%)	-	6 (14.0%)	
3	40 (26.3%)	-	12 (27.9%)	
4	61 (47.4%)	218 (92.4%)	22 (51.2%)	
Recurrence rate (within 2 yr)	69/142 (48.6%)	91/236 (38.6%)	4/43 (9.30%)	

[1]Continuous variables are presented as mean values ± standard deviation or percentage of cases.

[2]Values in public HBV-HCC and HCV-HCC were obtained by modifying previously published tables [3,4].

[3]*p*-values were calculated using χ^2 test.
