# Supplementary material for: Recurrence-associated pathways in hepatitis B virus-positive hepatocellular carcinoma
Source: BMC Genomics. 2015 Apr 10;16(1):279. doi: 10.1186/s12864-015-1472-x (PMC4448317; doi:10.1186/s12864-015-1472-x)
Supplement: Additional file 3: Table S3. — Pathways associated with recurrence in each HCC dataset. [file 12864_2015_1472_MOESM3_ESM.pdf]

Table S3. Pathways associated with recurrence in each HCC dataset.

	HBV-HCC					Public HBV-HCC					Public HCV-HCC				
**Pathway**	**PCA[1]**	**Weighted PCA[2]**	**FDR[3]**	**ROC AUC[4]**	**LS/KS**	**PCA**	**Weighted PCA**	**FDR**	**ROC AUC**	**LS/KS**	**PCA**	**Weighted PCA**	**FDR**	**ROC AUC**	**LS/KS**
ABC transporters	0.0650	0.0713	0.082		0.221/0.554	0.0549	0.0137	0.029		0.037/0.081	**0.0081**	0.0113	0.086	0.861	0.122/0.735
Adherens junction	0.2350	0.1332	0.06		0.144/0.296	**0.0021**	0.0064	0.004	0.687	0.094/0.204	0.0266	0.029	0.13		0.199/0.171
Amyotrophic lateral sclerosis	**0.0050**	0.0135	0.106	0.725	0.043/0.343	0.0111	0.0316	0.017		0.095/0.299	0.0464	0.0487	0.146		0.585/0.718
Apoptosis	**0.0088**	0.021	0.048	0.675	0.055/0.044	**0.0003**	0.0045	0.002	0.747	0.002/0.002	0.0185	0.0169	0.07		0.406/0.652
Arrhythmogenic right ventricular cardiomyopathy ARVC	0.2005	0.1769	0.091		0.347/0.427	**0.0032**	0.0126	0.006	0.685	0.02/0.186	0.0207	0.0209	0.119		0.482/0.412
AT1R pathway	0.0438	0.0571	0.038		0.045/0.15	**0.0017**	0.0064	0.005	0.746	0.004/0.051	0.0463	0.0635	0.158		0.501/0.953
Axon guidance (KEGG)	**0.004**	0.0133	0.015	0.677	0.008/0.011	**0.0003**	0.0013	0.008	0.700	0.068/0.452	**0.0062**	0.0061	0.094	0.877	0.472/0.801
Axon guidance (Reactome)	**0.0073**	0.0199	0.025	0.705	0.031/0.099	**0.0007**	0.0029	0.005	0.736	0.062/0.469	**0.0065**	0.0053	0.084	0.810	0.528/0.885
Calcium signaling pathway	**0.0098**	0.0892	0.067	0.691	0.255/0.863	**0.0002**	0.0019	0.006	0.738	0.009/0.028	0.0123	0.0072	0.105		0.549/0.927
CDT1 association with the CDC6 ORC origin comple	0.1074	0.1034	0.075		0.25/0.558	**0.0023**	0.005	0.03	0.726	0.014/0.101	0.0617	0.036	0.154		0.683/0.628
Cell adhesion molecules cams	**0.0035**	0.0111	0.062	0.677	0.156/0.561	**0.0066**	0.0177	0.018	0.711	0.095/0.202	0.0175	0.013	0.152		0.756/0.184
Cell death signalling via NRAGE, NRIF and NADE	0.0319	0.0563	0.097		0.226/0.292	**0.003**	0.0121	0.006	0.653	0.052/0.58	0.0225	0.02	0.124		0.631/0.67
Cell junction organization	**0.0068**	0.0126	0.084	0.686	0.031/0.16	**0.0075**	0.0296	0.003	0.684	0.009/0.02	0.0193	0.0167	0.111		0.749/0.822
Cell surface interactions at the vascular wall	**0.0055**	0.0199	0.053	0.726	0.053/0.27	**0.0018**	0.0151	0.018	0.705	0.023/0.031	0.0122	0.0063	0.129		0.632/0.982
Chemokine receptors bind chemokines	**0.0057**	0.0408	0.044	0.658	0.05/0.512	**0.0004**	0.0013	0.001	0.743	0.009/0.015	0.0658	0.0815	0.204		0.676/0.795
Chemokine signaling pathway	**0.0026**	0.0237	0.019	0.716	0.023/0.027	**0.0043**	0.0007	0.003	0.745	0.002/0.01	0.0153	0.0168	0.11		0.38/0.437
Class A1 rhodopsin like receptors	**0.0022**	0.0047	0.019	0.680	0.022/0.222	**0.0002**	0.0147	0.004	0.695	0.001/0.017	0.0122	0.0102	0.112		0.678/0.467
Cytokine cytokine receptor interaction	**0.0022**	0.0134	0.042	0.695	0.057/0.227	**0.0019**	0.0107	0.002	0.694	0.012/0.006	0.0172	0.0233	0.116		0.719/0.923
Dilated cardiomyopathy	0.0779	0.1382	0.074		0.276/0.545	**0.0015**	0.0171	0.004	0.686	0.004/0.018	0.0168	0.0166	0.149		0.59/0.366
DNA repair	0.042	0.0577	0.056		0.451/0.945	**0.0027**	0.0085	0.01	0.704	0.01/0.038	0.0105	0.0065	0.113		0.757/0.519
DNA replication pre initiation	0.0407	0.1076	0.074		0.233/0.524	**0.0023**	0.0044	0.02	0.728	0.015/0.009	0.0443	0.0271	0.143		0.746/0.558
Downstream events in GPCR signaling	**0.0012**	0.0039	0.027	0.740	0.065/0.347	**0.0001**	0.0067	0.002	0.720	0.003/0.016	**0.0055**	0.0031	0.086	0.961	0.629/0.411
Downstream signaling of activated FGFR	0.1913	0.1598	0.088		0.204/0.556	**0.0007**	0.0051	0.002	0.707	0.002/0.013	0.1596	0.1596	0.135		0.903/0.728
Endocytosis	**0.0074**	0.0315	0.031	0.629	0.273/0.198	**0.0017**	0.0019	0.004	0.696	0.004/0.033	**0.007**	0.0081	0.089	0.852	0.493/0.678
ERBB signaling pathway	**0.002**	0.0082	0.048	0.729	0.086/0.413	**0.0017**	0.0075	0.005	0.680	0.011/0.031	0.0315	0.0209	0.103		0.776/0.731
Fc gamma R-mediated phagocytosis	**0.0074**	0.0117	0.042	0.676	0.067/0.294	0.0139	0.0413	0.019		0.079/0.102	0.0219	0.0233	0.13		0.348/0.48
Focal adhesion	**0.004**	0.0316	0.013	0.656	0.015/0.035	**0.0007**	0.0096	0.009	0.738	0.03/0.124	0.01	0.0063	0.09		0.571/0.996
Formation of platelet plug	**0.0012**	0.0124	0.021	0.718	0.006/0.016	**0.0037**	0.0485	0.002	0.711	0.012/0.004	**0.0089**	0.0231	0.103	0.707	0.622/0.853
Further platelet releasate	**0.0051**	0.0145	0.008	0.680	0/0.008					0.521/0.889	0.0539	0.0601	0.26		0.35/0.412
G alpha i signalling events	**0.0049**	0.0045	0.007	0.660	0.023/0.161	0.0138	0.0033	0.003		0.029/0.144	0.0165	0.0198	0.106		0.653/0.4
G alpha q signalling events	**0.0051**	0.0144	0.064	0.680	0.168/0.577	**0.0001**	0.003	0.003	0.725	0.001/0.014	0.019	0.0171	0.098		0.659/0.263
G alpha s signalling events	0.0364	0.0284	0.028		0.05/0.254	**0.0024**	0.0125	0.006	0.671	0.023/0.055	0.0188	0.0185	0.11		0.4/0.931
Gap junction	0.0959	0.0423	0.055		0.153/0.587	**0.001**	0.0086	0.006	0.653	0.018/0.073	0.0181	0.0149	0.146		0.508/0.99
Gleevec pathway	**0.0055**	0.0182	0.016	0.722	0.002/0.092	0.0561	0.0165	0.03		0.01/0.019	0.081	0.0926	0.133		0.403/0.268
Glucose and other sugar slc transporters	**0.0057**	0.0159	0.074	0.676	0.227/0.464	0.0348	0.0318	0.013		0.016/0.041	0.011	0.0129	0.126		0.419/0.81
Glucose transport	0.1015	0.1562	0.114		0.819/0.615	0.0139	0.0195	0.01		0.013/0.008	**0.0051**	0.0052	0.143	0.763	0.5/0.961

Pathway	PCA	Weighted PCA[2]	FDR	ROC AUC	LS/KS	PCA	Weighted PCA[2]	FDR	ROC AUC	LS/KS	PCA	Weighted PCA[2]	FDR	ROC AUC	LS/KS
Glycerophospholipid metabolism	0.1388	0.1397	0.07		0.361/0.881	**0.003**	0.0109	0.006	0.688	0/0	0.0475	0.046	0.12		0.908/0.547
Glycosphingolipid biosynthesis ganglio series	0.1407	0.1407	0.185		0.227/0.267					0.891/0.244	**0.0005**	0.0012	0.087	0.856	0.006/0.216
Glycosphingolipid biosynthesis globo series	0.024	0.0362	0.03		0.009/0.205	0.0775	0.1131	0.069		0.13/0.392	**0.0011**	0.001	0.053	0.872	0.008/0.284
GPCR ligand binding	**0.002**	0.0051	0.005	0.708	0.038/0.187	**0.0001**	0.0103	0.004	0.725	0.007/0.019	**0.0089**	0.0093	0.084	0.912	0.652/0.669
Hematopoietic cell lineage	0.0638	0.1	0.142		0.659/0.559	**0.0018**	0.018	0.008	0.692	0.039/0.076	0.0414	0.0537	0.142		0.665/0.852
Hemostasis	**0.0005**	0.0098	0.016	0.736	0.014/0.029	**0.0031**	0.0641	0.007	0.711	0.015/0.006	**0.009**	0.0066	0.101	0.585	0.709/0.703
HIF pathway	**0.0089**	0.0208	0.019	0.674	0/0.004	**0.0067**	0.019	0.02	0.656	0.004/0.038	0.1967	0.1967	0.285		0.762/0.807
HIV infection	**0.0091**	0.0516	0.036	0.668	0.427/0.5	**0.0034**	0.0037	0.006	0.678	0.011/0.063	**0.0059**	0.0027	0.091	0.705	0.31/0.705
Host interactions of HIV factors	**0.0023**	0.0412	0.049	0.660	0.341/0.642	**0.0024**	0.0049	0.008	0.690	0.003/0.045	0.0104	0.0049	0.121		0.291/0.559
Hypertrophic cardiomyopathy	0.195	0.2127	0.075		0.459/0.895	**0.0025**	0.0182	0.007	0.688	0.008/0.039	0.0185	0.0282	0.121		0.658/0.498
Immunoregulatory interactions between a lymphoid and a non lymphoid cell	**0.01**	0.0312	0.015	0.622	0.045/0.055	0.0159	0.0413	0.035		0.065/0.122	0.0336	0.0433	0.194		0.359/0.403
Innate immunity signaling	**0.0081**	0.0292	0.021	0.690	0.02/0.056	0.0403	0.006	0.009		0.072/0.503	**0.009**	0.0071	0.094	0.860	0.57/0.676
Inorganic cation anion SLC transporters	0.0581	0.0975	0.089		0.442/0.911	**0.0043**	0.0208	0.006	0.657	0.001/0.009	**0.0075**	0.0069	0.104	0.869	0.636/0.669
Insulin signaling pathway	**0.009**	0.0403	0.008	0.652	0.095/0.171	**0.0047**	0.0137	0.01	0.668	0.005/0.026	0.0122	0.0159	0.097		0.717/0.704
Integrin cell surface interactions	**0.0031**	0.0268	0.009	0.720	0.016/0.04	**0.0023**	0.0115	0.003	0.725	0.008/0.021	0.0274	0.028	0.129		0.845/0.134
Integrin pathway	0.1287	0.0409	0.037		0.002/0.026	**0.0035**	0.0113	0.01	0.7317	0.014/0.096	0.0209	0.0283	0.129		0.408/0.763
IRS related events	0.2007	0.2175	0.077		0.267/0.372	**0.0012**	0.0103	0.007	0.6965	0.037/0.376	0.0318	0.0364	0.11		0.823/0.601
Keratinocyte pathway	**0.0064**	0.0223	0.019	0.671	0.032/0.272	0.012	0.0221	0.01		0.005/0.099	0.0268	0.0301	0.168		0.203/0.292
Leishmania infection	**0.0042**	0.0205	0.01	0.730	0.006/0.014	0.0536	0.011	0.023		0.192/0.48	0.0247	0.0254	0.151		0.772/0.427
Leukocyte transendothelial migration	0.0496	0.0517	0.067		0.078/0.524	0.0214	0.009	0.006		0.011/0.02	**0.0082**	0.0109	0.131	0.931	0.437/0.629
Lysosome	**0.0026**	0.0375	0.06	0.694	0.277/0.891	**0.0037**	0.021	0.003	0.684	0.096/0.201	**0.0037**	0.0047	0.08	0.863	0.805/0.693
M G1 transition	0.1161	0.1106	0.083		0.248/0.506	**0.0019**	0.0036	0.029	0.726	0.011/0.011	0.0445	0.0268	0.156		0.62/0.763
MAP kinases activation in TLR cascade	**0.0082**	0.0195	0.007	0.681	0.012/0.105	0.0296	0.057	0.011		0.699/0.556					0.555/0.878
MAPK signaling pathway	**0.003**	0.011	0.011	0.702	0.032/0.044	**0.0006**	0.0119	0.001	0.718	0.018/0.085	0.0119	0.0117	0.076	0.838	0.857/0.712
Melanogenesis	0.1073	0.0794	0.088		0.267/0.623	**0.003**	0.0231	0.003	0.633	0.033/0.137	0.0158	0.0149	0.085		0.42/0.638
Membrane trafficking	**0.0067**	0.0868	0.064	0.669	0.226/0.372	**0.0036**	0.0065	0.013	0.670	0.21/0.804	0.0171	0.0146	0.096		0.637/0.584
MET pathway	0.0116	0.013	0.009		0.002/0.028	**0.0026**	0.0116	0.006	0.718	0.009/0.015	0.0656	0.0555	0.167		0.795/0.93
Metabolism of amino acids	**0.007**	0.0215	0.041	0.6597	0.368/0.728	0.0816	0.0428	0.011		0.006/0.005	0.0206	0.0177	0.11		0.874/0.287
Metabolism of lipids and lipoproteins	**0.0015**	0.0102	0.014	0.700	0.035/0.05	0.0517	0.0356	0.009		0.011/0.008	**0.0045**	0.0029	0.085	0.868	0.459/0.946
Metabolism of RNA	0.0181	0.0515	0.04		0.204/0.812	0.0113	0.038	0.011		0.018/0.045	**0.0078**	0.0042	0.117	0.800	0.296/0.405
Natural killer cell mediated cytotoxicity	**0.0021**	0.0114	0.044	0.696	0.229/0.516	**0.0006**	0.0031	0.011	0.726	0.024/0.017	0.0208	0.0298	0.145		0.255/0.158
Neuroactive ligand receptor interaction	0.1862	0.2184	0.06		0.495/0.509	**0.0003**	0.0068	0.004	0.711	0.04/0.11	0.0128	0.0118	0.104		0.848/0.484
Neurotrophin signaling pathway	**0.0028**	0.0183	0.034	0.679	0.269/0.843	**0.003**	0.0101	0.006	0.685	0.053/0.215	0.0137	0.016	0.087		0.523/0.881
Opioid signalling	0.1433	0.0689	0.046		0.018/0.021	**0.0078**	0.0395	0.005	0.658	0.055/0.135	**0.0091**	0.0097	0.126	0.539	0.194/0.375
Orc1 removal from chromatin	0.0482	0.0978	0.046		0.137/0.242	**0.002**	0.0042	0.031	0.726	0.012/0.038	0.0539	0.033	0.163		0.727/0.506
P75 NTR receptor mediated signalling	0.0207	0.0388	0.045		0.235/0.082	**0.0027**	0.0118	0.008	0.682	0.008/0.089	0.0155	0.0107	0.092		0.679/0.46
PAR1 pathway	0.1196	0.1288	0.075		0.289/0.345	**0.004**	0.0109	0.006	0.645	0.033/0.16	0.0178	0.0111	0.175		0.434/0.938
Pathways in cancer	**0.0026**	0.0281	0.008	0.726	0.027/0.159	**0.0012**	0.0262	0	0.692	0.003/0.003	**0.0059**	0.0028	0.064	0.796	0.577/0.708

Pathway	PCA	Weighted PCA[2]	FDR	ROC AUC	LS/KS	PCA	Weighted PCA[2]	FDR	ROC AUC	LS/KS	PCA	Weighted PCA[2]	FDR	ROC AUC	LS/KS
Peptide ligand binding receptors	0.0267	0.0063	0.046		0.005/0.129	**0.0026**	0.0074	0.006	0.661	0.006/0.015	0.0302	0.0428	0.125		0.823/0.672
PI3K cascade	0.1965	0.1908	0.099		0.161/0.4	**0.0013**	0.01	0.006	0.700	0.003/0.041	0.1613	0.1613	0.171		0.875/0.778
Platelet activation	**0.0012**	0.0083	0.017	0.727	0.012/0.034	**0.0093**	0.042	0.016	0.693	0.024/0.009	**0.0081**	0.0227	0.101	0.694	0.58/0.716
Platelet de-granulation	**0.0012**	0.0053	0.009	0.699	0.002/0.006	0.0111	0.0764	0.016		0.014/0.005	0.0261	0.0424	0.123		0.563/0.613
Processing of capped intron containing pre mRNA	**0.0095**	0.0457	0.046	0.670	0.182/0.491	0.0505	0.1329	0.013		0.027/0.029	0.01	0.0071	0.091		0.622/0.746
Prostate cancer	0.0645	0.0597	0.069		0.231/0.603	**0.0026**	0.0042	0.008	0.697	0.028/0.167	0.0203	0.0138	0.095		0.472/0.963
PYK2 pathway	**0.0035**	0.0078	0.004	0.659	0.023/0.244	0.0143	0.0362	0.02		0.013/0.142	0.0531	0.0612	0.217		0.387/0.325
Pyrimidine metabolism	0.0456	0.0334	0.036		0.098/0.327	**0.0041**	0.0159	0.009	0.647	0.013/0.107	0.013	0.0203	0.102		0.896/0.431
Pyruvate metabolism	**0.0077**	0.0321	0.011	0.674	0.022/0.046	0.1295	0.0378	0.036		0.002/0.001	0.0328	0.0203	0.222		0.912/0.231
Pyruvate metabolism and TCA cycle	0.0148	0.023	0.146		0.065/0.623	0.085	0.0497	0.043		0.086/0.273	**0.003**	0.0036	0.17	0.945	0.526/0.934
Regulation of actin cytoskeleton	**0.0025**	0.0294	0.037	0.677	0.04/0.186	**0.0004**	0.0051	0.007	0.692	0.19/0.7	**0.0098**	0.0093	0.091	0.812	0.521/0.854
Regulation of glucokinase by glucokinase regulatory protein	0.1224	0.1714	0.117		0.801/0.529	0.0294	0.0353	0.023		0.018/0.039	**0.0065**	0.0062	0.186	0.763	0.35/0.616
Renal cell carcinoma	**0.0072**	0.0322	0.056	0.670	0.071/0.173	**0.0029**	0.0187	0.005	0.675	0.017/0.064	0.0212	0.0207	0.106		0.625/0.832
REV mediated nuclear export of HIV1 RNA	0.0975	0.1422	0.097		0.825/0.835	0.0409	0.0253	0.03		0.014/0.033	**0.0052**	0.0053	0.154	0.763	0.42/0.665
RHO GTPase cycle	0.026	0.0332	0.06		0.105/0.388	**0.0019**	0.0121	0.004	0.653	0.094/0.221	0.0112	0.0071	0.105		0.709/0.4
RIG I like receptor signaling pathway	0.0602	0.0887	0.068		0.122/0.1	**0.0007**	0.0013	0.009	0.730	0.028/0.221	0.0229	0.0289	0.114		0.349/0.845
RNA degradation	**0.0071**	0.021	0.07	0.679	0.086/0.599	0.0253	0.0425	0.014		0.247/0.68	0.0123	0.0132	0.117		0.402/0.356
S phase	**0.01**	0.0786	0.053	0.645	0.273/0.744	**0.0025**	0.0101	0.023	0.682	0.039/0.043	0.0264	0.0178	0.129		0.806/0.337
SCF beta TRCP mediated degradation of EMI1	0.1029	0.1103	0.092		0.211/0.348	**0.0024**	0.0035	0.03	0.716	0.011/0.091	0.0583	0.0398	0.169		0.749/0.594
Signaling by EGFR	0.1377	0.1934	0.125		0.368/0.367	**0.002**	0.0094	0.008	0.691	0.018/0.105	0.0536	0.0565	0.124		0.727/0.996
Signaling by WNT	0.1633	0.0324	0.074		0.128/0.242	**0.0025**	0.0042	0.016	0.721	0.009/0.192	0.0342	0.0449	0.137		0.647/0.859
Signaling in immune system	**0.0018**	0.0133	0.026	0.717	0.048/0.118	**0.0002**	0.0044	0.01	0.713	0.029/0.084	**0.0054**	0.0018	0.111	0.920	0.626/0.634
Signalling by NGF	**0.0031**	0.0377	0.034	0.701	0.12/0.234	**0.0006**	0.0122	0.002	0.723	0.005/0.014	**0.0059**	0.0076	0.072	0.635	0.649/0.509
SLC mediated transmembrane transport	**0.0029**	0.0195	0.053	0.718	0.253/0.516	**0.0099**	0.009	0.006	0.674	0.003/0.007	**0.0049**	0.0051	0.1	0.811	0.489/0.983
Sphingolipid metabolism	0.1729	0.1729	0.122		0.522/0.686	0.062	0.0359	0.041		0.045/0.269	**0.0055**	0.004	0.073	0.844	0.034/0.281
Striated muscle contraction	0.1193	0.1193	0.18		0.423/0.918	**0.0049**	0.0088	0.019	0.694	0.007/0.123	0.0486	0.0298	0.264		0.174/0.202
Synthesis of DNA	0.0594	0.0898	0.063		0.211/0.606	**0.0009**	0.005	0.021	0.711	0.04/0.067	0.06	0.0404	0.147		0.887/0.238
TGF beta signaling pathway	0.2864	0.2909	0.089		0.136/0.077	**0.0009**	0.01	0.005	0.714	0.013/0.022	0.0146	0.0201	0.094		0.522/0.686
Tight junction	**0.0074**	0.0248	0.052	0.727	0.291/0.829	**0.0044**	0.017	0.006	0.693	0.02/0.185	0.0103	0.0107	0.096		0.271/0.159
Toll like receptor 3 cascade	**0.0069**	0.0256	0.014	0.703	0.007/0.02	**0.0048**	0.0148	0.011	0.628	0.405/0.403	0.0155	0.0162	0.118		0.297/0.853
Toll like receptor signaling pathway	**0.0019**	0.0101	0.005	0.698	0.004/0.029	**0.0032**	0.0012	0.006	0.624	0.035/0.322	0.0291	0.0276	0.106		0.857/0.582
Toll receptor cascades	**0.0077**	0.0307	0.012	0.693	0.008/0.051	**0.0012**	0.0033	0.005	0.688	0.051/0.591	0.015	0.011	0.118		0.54/0.942
TPO pathway	**0.0086**	0.0486	0.056	0.694	0.012/0.033	**0.0046**	0.0151	0.009	0.643	0.002/0.002	0.0679	0.0732	0.109		0.688/0.833
TRAF6 mediated induction of the antiviral cytokine IFN alpha beta cascade	**0.0088**	0.029	0.016	0.668	0.009/0.043	**0.0078**	0.014	0.013	0.650	0.564/0.295	0.0277	0.0297	0.132		0.592/0.945
Transmembrane transport of small molecules	**0.0015**	0.0089	0.033	0.749	0.248/0.475	**0.0049**	0.0495	0.004	0.668	0/0	**0.0032**	0.0029	0.079	0.883	0.477/0.975
Transport of the SLBP independent mature mRNA	0.055	0.0835	0.082		0.547/0.936	0.0578	0.0366	0.032		0.068/0.109	**0.0049**	0.0048	0.172	0.763	0.469/0.937
Trka signalling from the plasma membrane	0.0547	0.0481	0.029		0.062/0.223	**0.0014**	0.0126	0.004	0.7001	0.006/0.005	0.0215	0.0305	0.084		0.826/0.398
Ubiquitin mediated proteolysis	0.2615	0.2045	0.051		0.269/0.122	**0.0025**	0.0128	0.002	0.680	0.01/0.027	0.014	0.0071	0.081		0.738/0.809

Pathway	PCA	Weighted PCA[2]	FDR	ROC AUC	LS/KS	PCA	Weighted PCA[2]	FDR	ROC AUC	LS/KS	PCA	Weighted PCA[2]	FDR	ROC AUC	LS/KS
Vascular smooth muscle contraction	**0.0059**	0.0523	0.022	0.666	0.096/0.109	**0.0027**	0.0271	0.008	0.696	0.011/0.017	0.0165	0.017	0.127		0.356/0.823
Vasopressin regulated water reabsorption	0.0556	0.0732	0.099		0.21/0.508	**0.0014**	0.0068	0.006	0.683	0.002/0.161	0.0527	0.0596	0.123		0.828/0.691
VEGF pathway	**0.0032**	0.0325	0.029	0.648	0.013/0.122	**0.0044**	0.0139	0.007	0.666	0.001/0.04	0.0929	0.0875	0.161		0.63/0.894
VEGF signaling pathway	0.1096	0.0864	0.041		0.06/0.082	**0.004**	0.0201	0.003	0.668	0.038/0.133	0.0487	0.0608	0.139		0.779/0.723
Vibrio cholerae infection	0.2015	0.0748	0.089		0.044/0.219	**0.0035**	0.0142	0.01	0.687	0.019/0.066	**0.007**	0.0059	0.111	0.808	0.449/0.469
VIF mediated degradation of apobec3g	0.1274	0.099	0.101		0.176/0.324	**0.0049**	0.0037	0.028	0.708	0.009/0.107	0.059	0.0356	0.158		0.638/0.906
WNT signaling pathway	0.0759	0.0703	0.049		0.145/0.125	0.0016	0.007	0.005	0.677	0.047/0.14	0.0129				0.438/0.749

Significant pathways (*p*-value<0.01) are highlighted with red bold characters.

[1]*p*-value was computed with permutation-based PCA using one optimal principal component.

[2]*p*-value was computed with permutation-based PCA using multiple principal components as weight.

[3]False discovery rate (FDR) was measured by implementing random sampling for permutation-based PCA .

[4]Receiver operating characteristic (ROC)-area under curve (AUC) was evaluated for early recurrence within two years of surgery.
