# Supplementary material for: Recurrence-associated pathways in hepatitis B virus-positive hepatocellular carcinoma
Source: BMC Genomics. 2015 Apr 10;16(1):279. doi: 10.1186/s12864-015-1472-x (PMC4448317; doi:10.1186/s12864-015-1472-x)
Supplement: Additional file 4: Figure S1. — Comparison of one-optimal PCA and weighted PCA methods. Log-rank p-values of 882 pathways calculated with the two methods were compared. One-optimal PCA used one principal component optimally associated with recurrence while weighted PCA used multiple principal component sex plaining over 80% variance in each dataset. [file 12864_2015_1472_MOESM4_ESM.pdf]

Figure S1

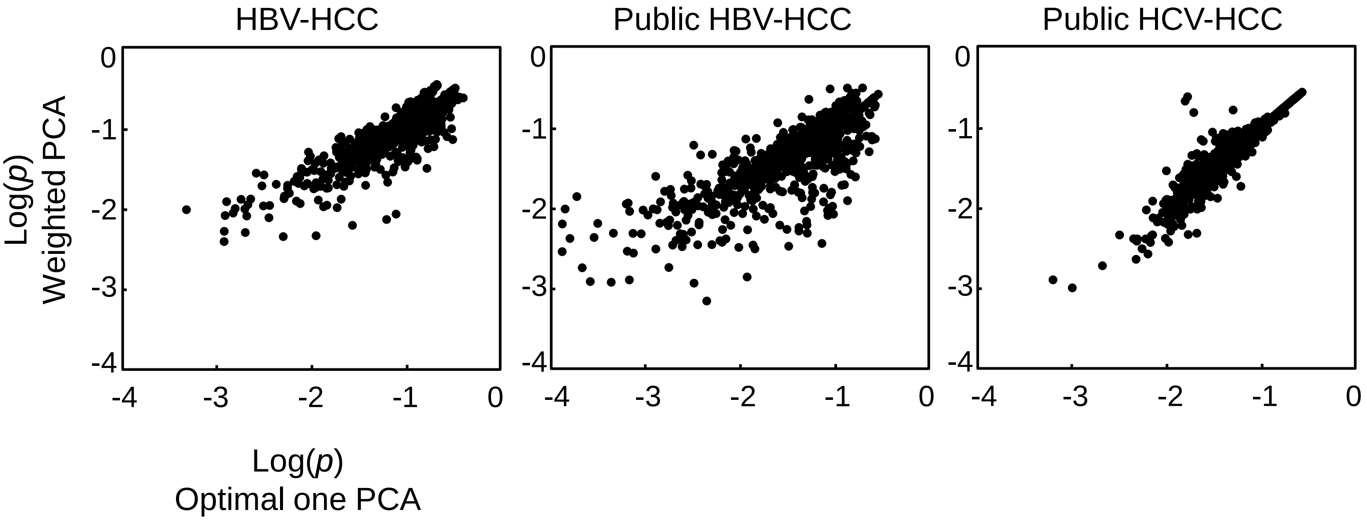

Figure S1. Comparison of one-optimal PCA and weighted PCA methods. Log-rank *p*-values of 882 pathways calculated with the two methods were compared. One-optimal PCA used one principal component optimally associated with recurrence while weighted PCA used multiple principal components explaining over 80% variance in each dataset.
