# Supplementary material for: Recurrence-associated pathways in hepatitis B virus-positive hepatocellular carcinoma
Source: BMC Genomics. 2015 Apr 10;16(1):279. doi: 10.1186/s12864-015-1472-x (PMC4448317; doi:10.1186/s12864-015-1472-x)
Supplement: Additional file 5: Figure S2. — Association of the axon guidance pathway with recurrence of HCC. Kaplan-Meier plot sand receiver operating characteristic (ROC) curve with the axon guidance pathway in HBV-HCC, public HBV-HCC and public HCV-HCC. For the Kaplan-Meier plot, samples were classified in to two subgroups based on the median value of principal components of the axon guidance pathway. [file 12864_2015_1472_MOESM5_ESM.pdf]

Figure S2

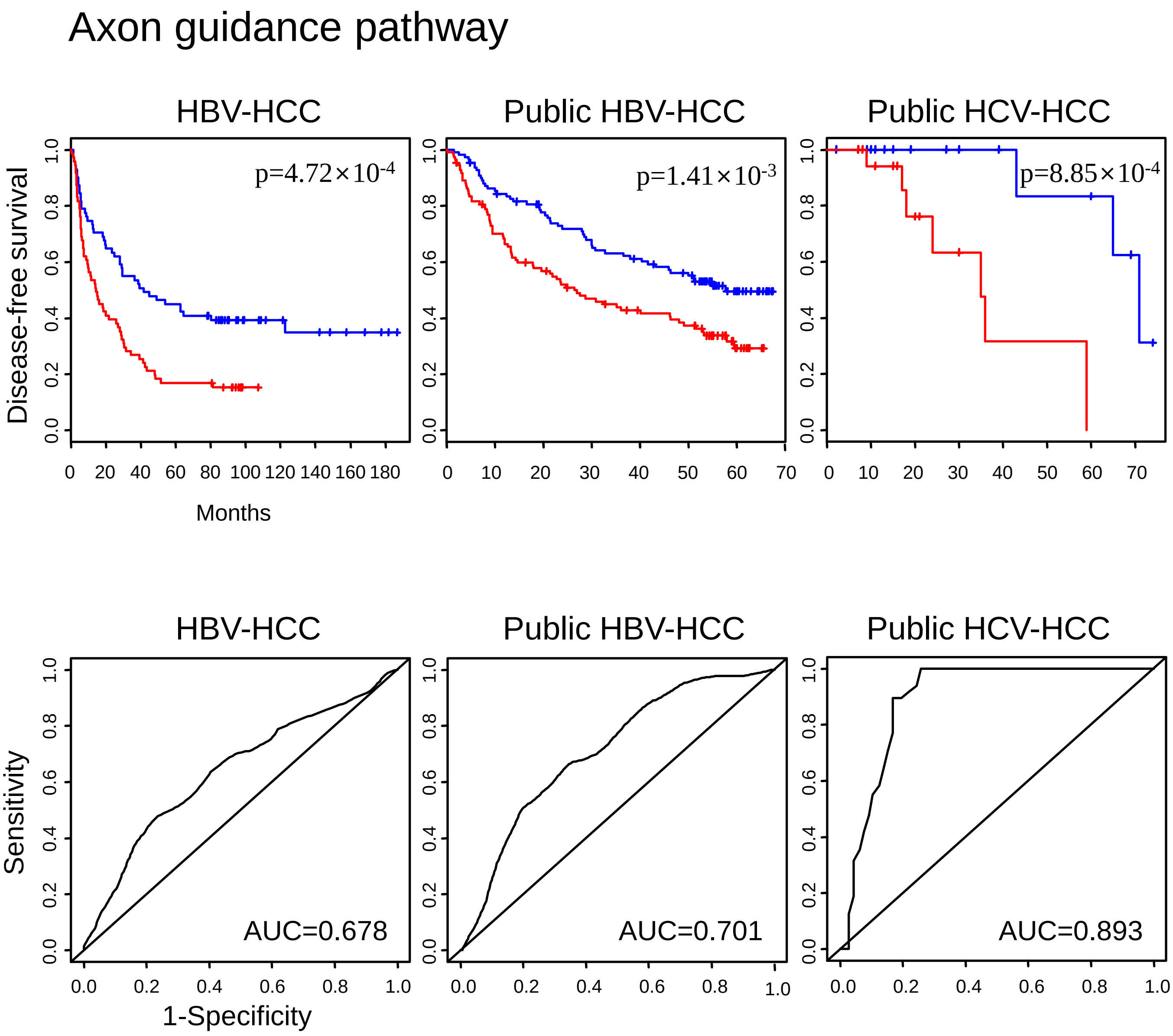

Figure S2. Association of the axon guidance pathway with recurrence of HCC. Kaplan-Meier plots and receiver operating characteristic (ROC) curve with the axon guidance pathway in HBV-HCC, public HBV-HCC and public HCV-HCC. For the Kaplan-Meier plot, samples were classified into two subgroups based on the median value of principal components of the axon guidance pathway.
