# Supplementary material for: Recurrence-associated pathways in hepatitis B virus-positive hepatocellular carcinoma
Source: BMC Genomics. 2015 Apr 10;16(1):279. doi: 10.1186/s12864-015-1472-x (PMC4448317; doi:10.1186/s12864-015-1472-x)
Supplement: Additional file 6: Figure S3. — Cluster analysis of recurrence-related pathways. (A) Dendrogram of clustering pattern measured from the matrix of principal components of 90 recurrence-associated pathways (p-value < 0.01) from public HBV-HCC. Samples were classified into two subgroups: low-risk and high-risk based on recurrence out come. Columns represent individual samples, and rows pathways. Red and green colors reflect high and low levels of optimal principal component scores, respectively, as indicated by scale bars. (B) Kaplan-Meier plots for recurrence rates of the low-and high-risk subgroups. (C) Cross-validation of the low-and high-risk subgroups using six different algorithms: compound covariate (CC), diagonal line ardiscriminant (DLD), 1-nearest neighbor (1-NN), 3-nearest neighbor (3-NN), nearest centroid (NC) and support vector machine (SVM). (D) ROC curve computed with CC validation algorithm for subgroup classification. [file 12864_2015_1472_MOESM6_ESM.pdf]

Figure S3

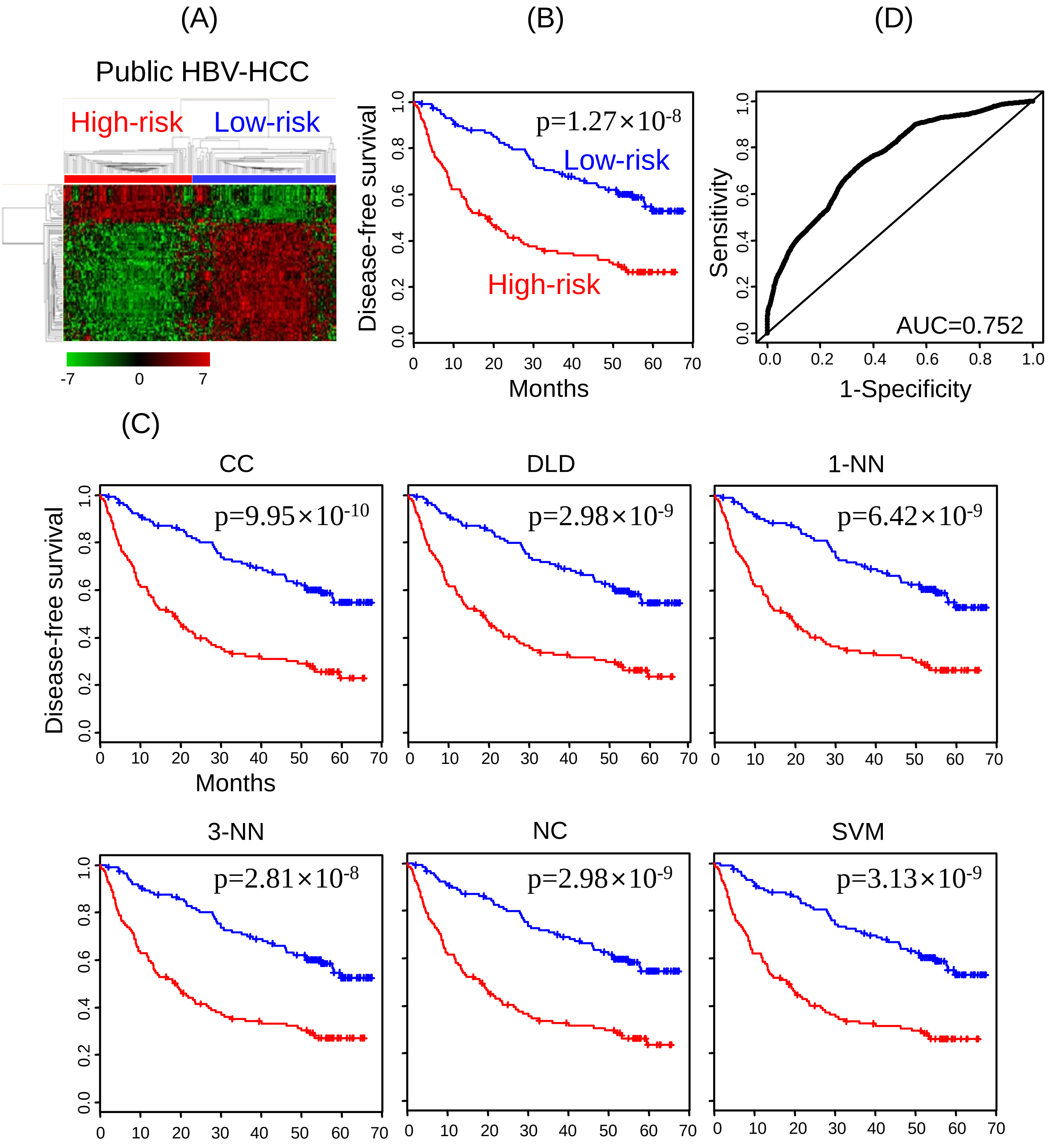

Figure S3. Cluster analysis of recurrence-related pathways. (A) Dendrogram of clustering pattern measured from the matrix of principal components of 90 recurrence-associated pathways (*p*-value<0.01) from public HBV-HCC. Samples were classified into two subgroups: low-risk and high-risk based on recurrence outcome. Columns represent individual samples, and rows pathways. Red and green colors reflect high and low levels of optimal principal component scores, respectively, as indicated by scale bars. **(B)** Kaplan-Meier plots for recurrence rates of the low- and high-risk subgroups. **(C)** Cross-validation of the low- and high-risk subgroups using six different algorithms: compound covariate (CC), diagonal linear discriminant (DLD), 1-nearest neighbor (1-NN), 3-nearest neighbor (3-NN), nearest centroid (NC) and support vector machine (SVM). **(D)** ROC curve computed with CC validation algorithm for subgroup classification.
