# Supplementary material for: Recurrence-associated pathways in hepatitis B virus-positive hepatocellular carcinoma
Source: BMC Genomics. 2015 Apr 10;16(1):279. doi: 10.1186/s12864-015-1472-x (PMC4448317; doi:10.1186/s12864-015-1472-x)
Supplement: Additional file 8: Figure S5. — Stratification of small tumor-size patients by recurrence-associated pathways. (A) Kaplan-Meier plot for recurrence rates in two subgroups of patients based on tumorsize ( 5 cm) in public HBV-HCC dataset. (B) Application of subgroup information determined from recurrence-associated pathways to small tumor-size patients (<5 cm) led to further stratification into two subgroups with different recurrence rates. [file 12864_2015_1472_MOESM8_ESM.pdf]

Figure S5

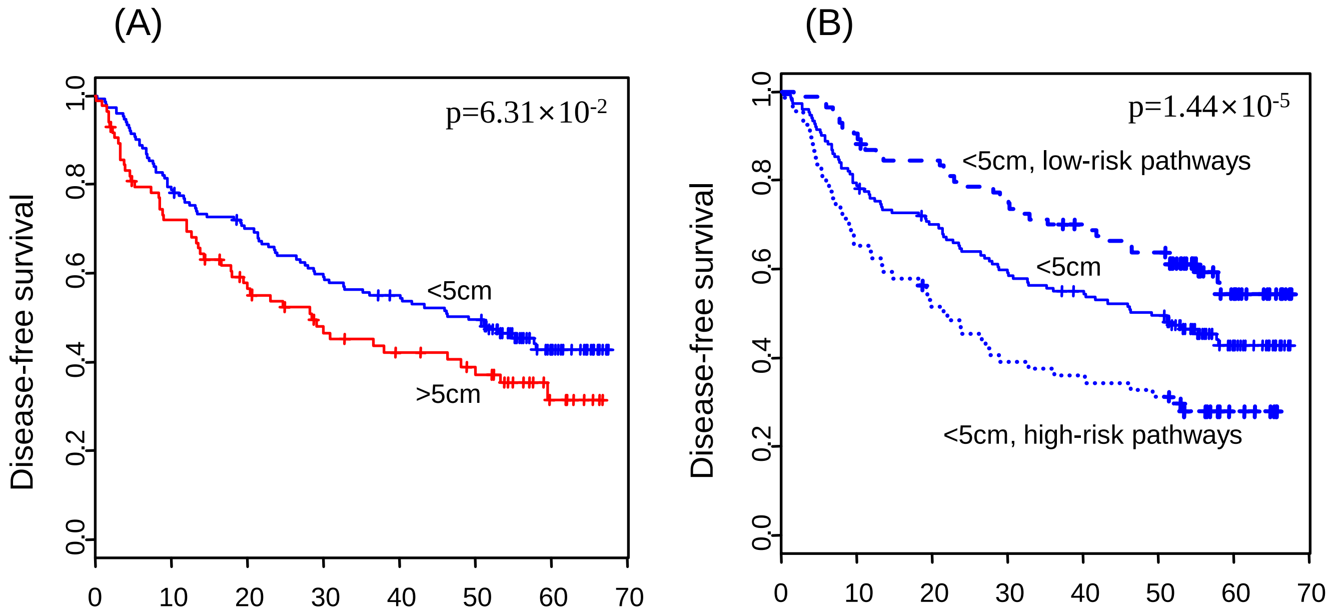

No. at Risk

<5cm	151	118	103	87	79	71	24	0
>5cm	84	57	42	32	27	22	7	0

Low risk	84	75	70	62	56	51	17	0
<5cm	151	118	103	87	79	71	24	0
High risk	67	43	33	25	23	20	7	0

Figure S5. Stratification of small tumor-size patients by recurrence-associated pathways. (A) Kaplan-Meier plot for recurrence rates in two subgroups of patients based on tumor size (< and > 5 cm) in public HBV-HCC dataset. **(B)** Application of subgroup information determined from recurrence-associated pathways to small tumor-size patients (<5 cm) led to further stratification into two subgroups with different recurrence rates.
