# Supplementary material for: Recurrence-associated pathways in hepatitis B virus-positive hepatocellular carcinoma
Source: BMC Genomics. 2015 Apr 10;16(1):279. doi: 10.1186/s12864-015-1472-x (PMC4448317; doi:10.1186/s12864-015-1472-x)
Supplement: Additional file 9: Figure S6. — Genes associated with lobular activity. A total of 206 genes were selected as differentially expressed (p < 0.001 with t-test) according to status of lobular activity. Among these genes, 23 were significantly associated with recurrence using the log-rank test (p < 0.01). [file 12864_2015_1472_MOESM9_ESM.pdf]

Figure S6

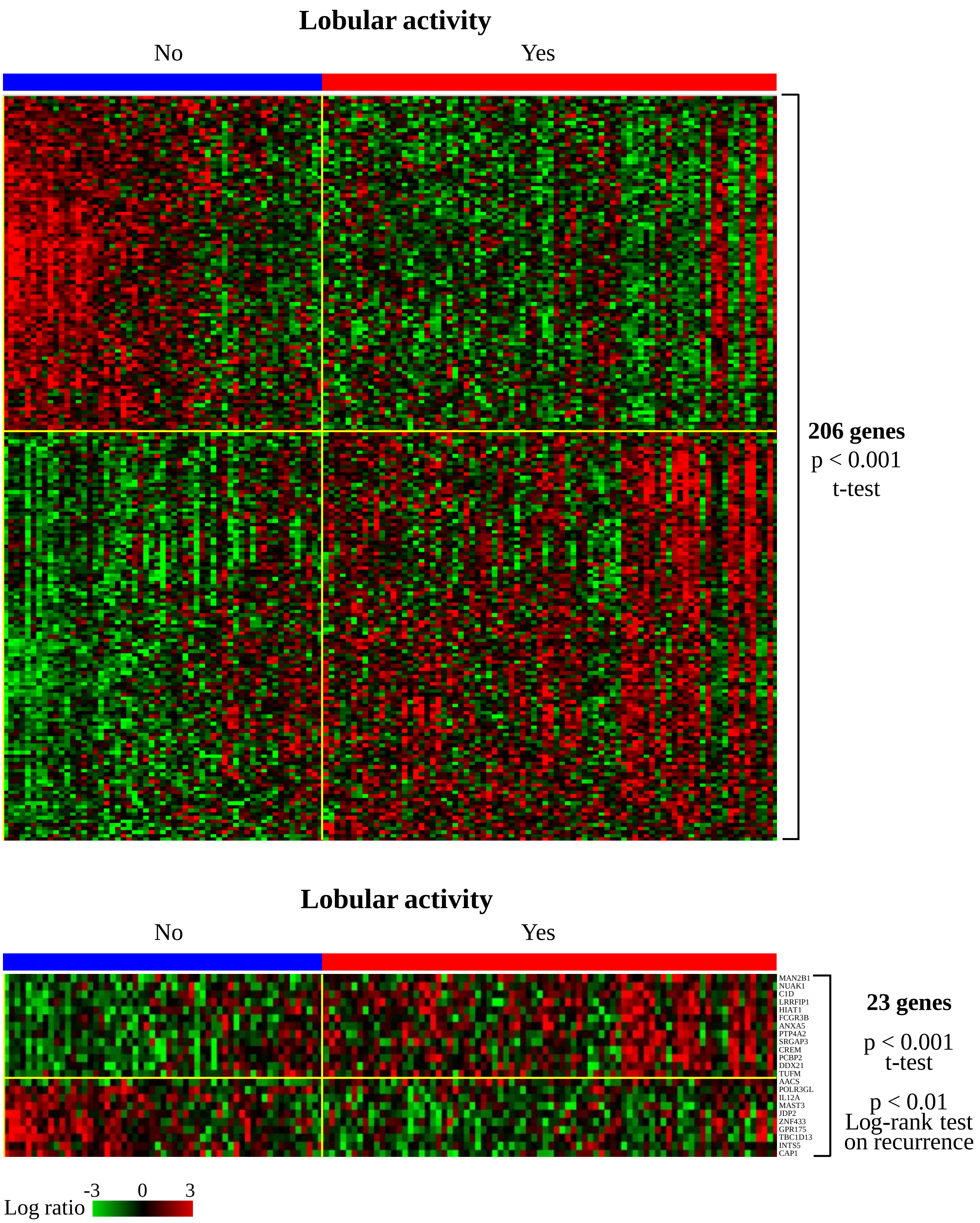

Figure S6. Genes associated with lobular activity. A total of 206 genes were selected as differentially expressed ($p<0.001$ with t-test) according to status of lobular activity. Among these genes, 23 were significantly associated with recurrence using the log-rank test ($p<0.01$).
