# Supplementary material for: Recurrence-associated pathways in hepatitis B virus-positive hepatocellular carcinoma
Source: BMC Genomics. 2015 Apr 10;16(1):279. doi: 10.1186/s12864-015-1472-x (PMC4448317; doi:10.1186/s12864-015-1472-x)
Supplement: Additional file 10: Figure S7. — Comparison of recurrence-associated and tumorigenic pathways. Recurrence-associated path ways identified in the present study were compared with those associated with tumorigenesis in HCC obtained from our prior analysis of the same public HBV and HCC datasets [12]. In total, (A) 64 recurrence-associated pathways from our HBV-HCC dataset (log-rank p-value < 0.01 in the testset) were compared with 84 tumorigenic pathways from another HBV-positive HCC dataset (error rate < 0.1), and (B) 32 recurrence-associated pathways from public HCV-HCC (log-rank p-value < 0.01 in the testset) compared with 138 tumorigenic pathways from the same dataset (error rate < 0.1). [file 12864_2015_1472_MOESM10_ESM.pdf]

Figure S7

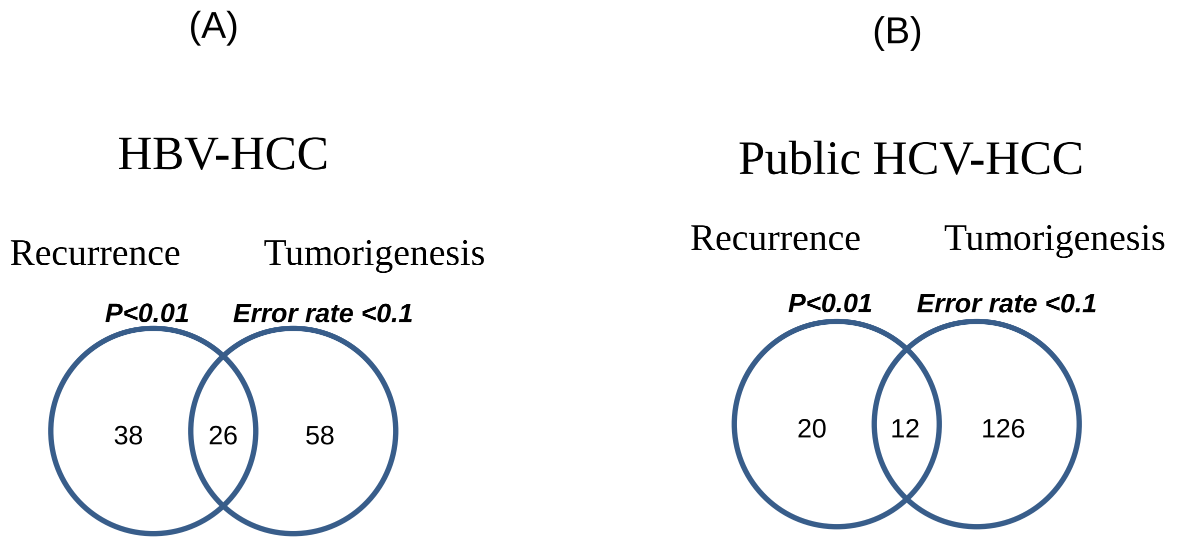

Figure S7. Comparison of recurrence-associated and tumorigenic pathways. Recurrence-associated pathways identified in the present study were compared with those associated with tumorigenesis in HCC obtained from our prior analysis of the same public HBV and HCC datasets [12]. In total, (A) 64 recurrence-associated pathways from our HBV-HCC dataset (log-rank p-value<0.01 in the test set) were compared with 84 tumorigenic pathways from another HBV-positive HCC dataset (error rate<0.1), and (B) 32 recurrence-associated pathways from public HCV-HCC (log-rank p-value<0.01 in the test set) compared with 138 tumorigenic pathways from the same dataset (error rate<0.1).
