# Supplementary material for: Recurrence-associated pathways in hepatitis B virus-positive hepatocellular carcinoma
Source: BMC Genomics. 2015 Apr 10;16(1):279. doi: 10.1186/s12864-015-1472-x (PMC4448317; doi:10.1186/s12864-015-1472-x)
Supplement: Additional file 11: Figure S8. — Distribution of recurrence-associated and tumorigenic pathways. [file 12864_2015_1472_MOESM11_ESM.pdf]

Figure S8

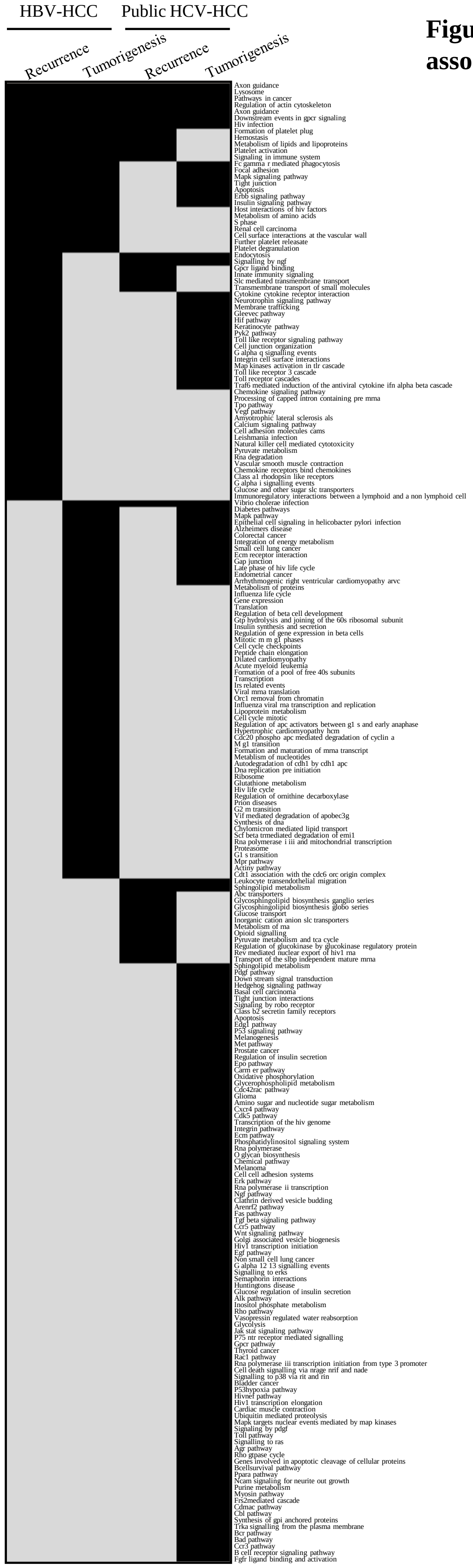

Figure S8. Distribution of recurrence-associated and tumorigenic pathways.
