# Supplementary material for: Recurrence-associated pathways in hepatitis B virus-positive hepatocellular carcinoma
Source: BMC Genomics. 2015 Apr 10;16(1):279. doi: 10.1186/s12864-015-1472-x (PMC4448317; doi:10.1186/s12864-015-1472-x)
Supplement: Additional file 12: Figure S9. — Comparison of patient classification. With previously reported prognosis-associated genes (Lee et al. [2], Hoshida et al. [8], public HBV-HCC [3] and public HCV-HCC u datasets), patient subclasses were determined using hierarchical clustering, and compared with subgroups obtained from our pathway-based method. Blue and red colors represent low-and high-risk subgroups, respectively, while white (public HBV-HCC) signifies the intermediate subgroup. Statistical significance was measured using Fisher’s exact test in comparison with pathway-based low-and high-risk subclasses. [file 12864_2015_1472_MOESM12_ESM.pdf]

Figure S9

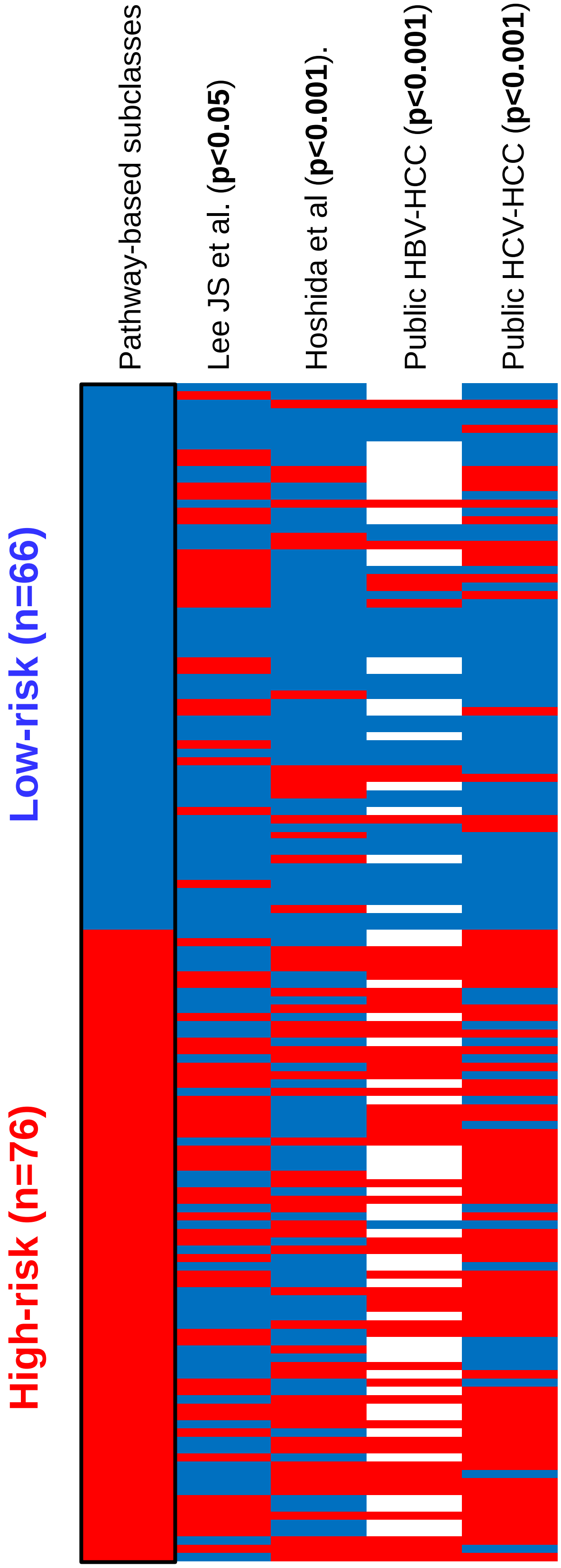

Figure S9. Comparison of patient classification. With previously reported prognosis-associated genes (Lee *et al*. [2], Hoshida *et al*. [8], public HBV-HCC [3] and public HCV-HCC [4] datasets), patient subclasses were determined using hierarchical clustering, and compared with subgroups obtained from our pathway-based method. Blue and red colors represent low- and high-risk subgroups, respectively, while white (public HBV-HCC) signifies the intermediate subgroup. Statistical significance was measured using Fisher's exact test in comparison with pathway-based low- and high-risk subclasses.
