# Supplementary material for: Recurrence-associated pathways in hepatitis B virus-positive hepatocellular carcinoma
Source: BMC Genomics. 2015 Apr 10;16(1):279. doi: 10.1186/s12864-015-1472-x (PMC4448317; doi:10.1186/s12864-015-1472-x)
Supplement: Additional file 13: Figure S10. — MA and QQ plots for assessing sample quality before normalization. The distribution of signal intensities of fluorescent probes was examined using MA and QQ plots. Older and newer samples indicate those collected before 1995 and after 2000, respectively. For QQ plot, we used the newest sample, S174, as a common reference. [file 12864_2015_1472_MOESM13_ESM.pdf]

Figure S10

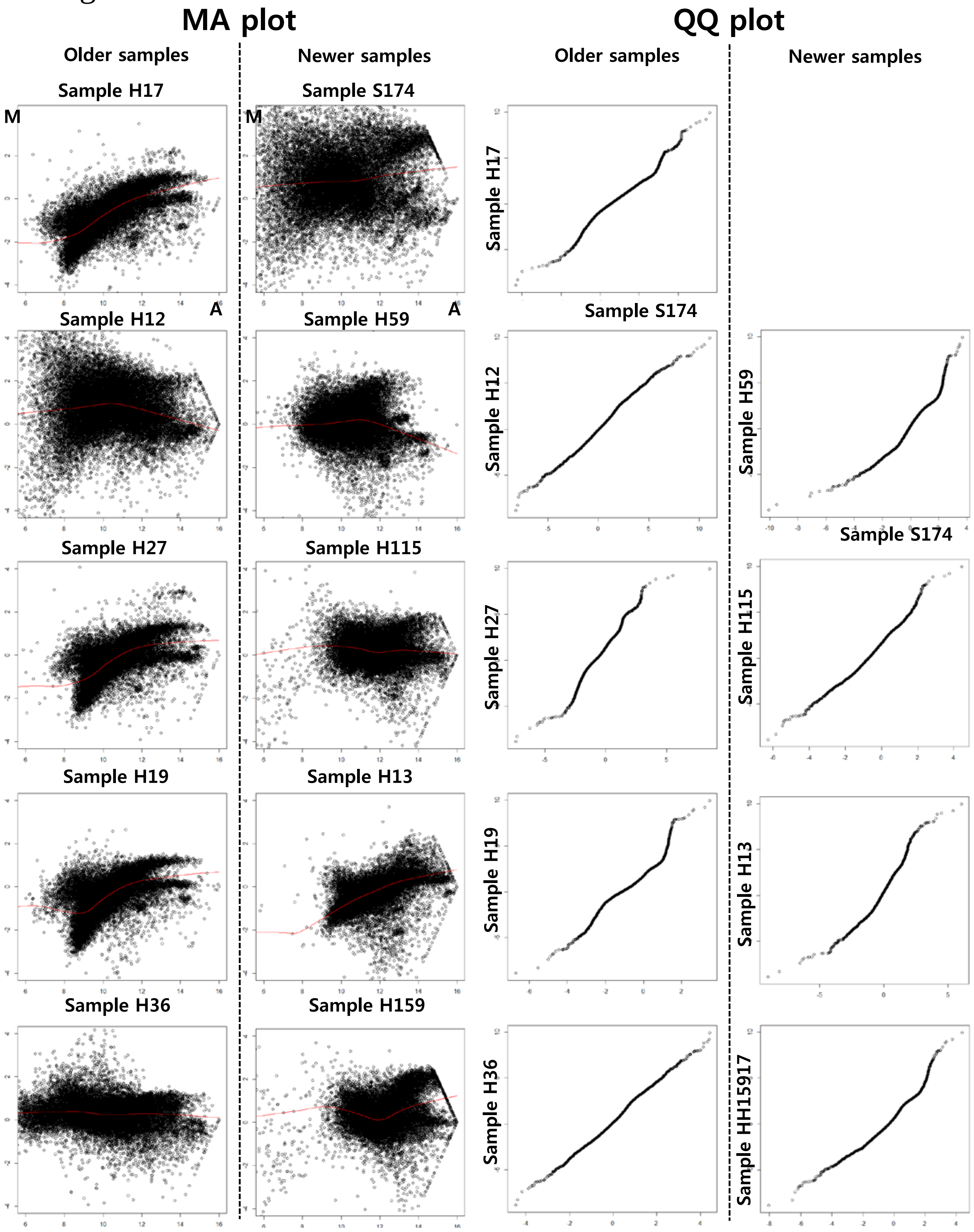

Figure S10. MA and QQ plots for assessing sample quality before normalization. The distribution of signal intensities of fluorescent probes was examined using MA and QQ plots. Older and newer samples indicate those collected before 1995 and after 2000, respectively. For QQ plot, we used the newest sample, S174, as a common reference.
